# Supplementary material for: Comparison of the effects of exergaming and balance training on dynamic postural stability during jump-landing in recreational athletes with chronic ankle instability
Source: PLoS One. 2024 Dec 16;19(12):e0314686. doi: 10.1371/journal.pone.0314686 (PMC11649137; doi:10.1371/journal.pone.0314686)
Supplement: S1 Text — (DOCX) [file pone.0314686.s002.docx]

**Comparison of the effects of exergaming and balance training on dynamic postural stability during jump-landing in recreational athletes with chronic ankle instability**

**Sadaf Sepasgozar**, **Roya Khanmohammadi**, **Zeinab Shiravi**

Department of Physical Therapy, Tehran University of Medical Sciences, Tehran, Iran.

**1. Introduction and statement of the problem**

The ankle is the most common injured joint in sports injuries, which includes 10 to 30% of sports injuries (1). According to the evidence and studies, ankle sprain is a permanent complication with long-term effects and can have destructive effects on the athlete's performance. Ankle sprains account for one-sixth of all time lost from participating in sports events (2). In other words, ankle sprains cause people to stay away from sports activities in addition to financial losses. Approximately 33% of people who experience lateral ankle sprains have chronic ankle instability (3, 4). Chronic instability refers to repeated ankle sprains after the initial sprain and deficits in neuromuscular control and mechanical stability (5, 6). A high percentage (23-61%) of athletes have chronic ankle instability, and football, basketball, and volleyball are the most frequently introduced sports teams in this injury (7).

In the mid-1960s, Freeman proposed that chronic ankle instability was strongly associated with poor postural control (8). Postural control is the ability to maintain balance by keeping the center of gravity on a supported surface. Maintaining postural control requires the integration of sensory information and the execution of appropriate motor responses (9). But in these people, due to damage to muscles and peripheral nervous structures, inhibition of the central nervous system and sensory reorganization, and neuromotor disorder, postural control and balance are disturbed (10). Postural control is very important and necessary for sports participation and performance, but it is reduced in people with ankle sprains or instability (11). The high prevalence of ankle injuries and the persistence of instability symptoms for long periods of time may cause disorders in the affected joint, which, if not properly treated, can cause significant problems in sports activities and daily functioning of a person (12). Therefore, improving posture control as one of the causes that predispose a person to frequent sprains and increasing injuries is of interest to researchers.

From a therapeutic point of view, various treatments have been proposed to improve balance and postural control in people with ankle instability. One of the treatments that has received attention in the last decade is exergame. Exergame consists of two words, sport and game, and it means using computer games to increase the level of physical activity (13). Exergame is a new technology that can be used to improve balance and is becoming a common intervention method worldwide (13). In this rehabilitation method, digital games are presented to the person and the user performs exercises to achieve the results of the game. These digital games can be without immersing the person in virtual space or with partial immersion or full immersion (14).

Although the effects of traditional exercises on improving the sense of balance have been proven, these programs have a weakness in that they are somewhat boring and cannot arouse the interest of the participants because in some cases it causes the person to complete the treatment period before achieving the desired results. But video games are engaging and entertaining (15, 16). Also, in traditional training programs, people are not encouraged to actively participate since they cannot have an immediate feedback, but in video games, visual and auditory feedbacks are frequently given to the person, which increases the participation rate of the person (17). The use of computer games can provide the possibility of personalizing treatment and documenting progress, and it can motivate and increase patients' participation (18-20). Also, since it does not require much supervision, it may reduce the workload of the therapist (21). It is also generally available commercially and can be used for home rehabilitation. Computer games can target several different cognitive and motor resources such as inhibiting irrelevant stimuli, decision making, concentration and strength and balance (22). Involvement of cognitive resources is one of the important benefits of this rehabilitation program. Studies have shown that in people with ankle instability, cognitive load can be considered as a risk factor in repeated sprains. In fact, there is a disorder in information processing in this category of people. Therefore, increasing movement skills while reducing dependence on conscious information processing and using double and multiple exercises and increasing the processing capacity of the central nervous system can be useful. The interconnectedness of required motor and cognitive resources in many tasks has led to the design of treatments in which cognitive and motor exercises are combined and used to improve postural control. In fact, researchers believe that combined motor and cognitive exercises are more effective than motor or cognitive exercises alone (23). Nowadays, video games are a promising option for combined motor and cognitive exercises (23). In addition, the system can provide a rehabilitation environment that allows users to repeat exercises while being motivated enough to perform a large number of exercises, all of which are important components in rehabilitation. are known (24). Another factor that improves balance after doing exercises with video games is the amount of feedback that the user receives while playing. Simultaneous feedback allows the user to focus more on their movements. In games, feedbacks are given to him based on performance and results, which can make him learn motor tasks better (25). Another advantage of video games is that during exercises, the user must constantly shift his weight in different directions at different speeds and intensities while maintaining the center of pressure at his functional reference level. These controlled movements are very similar to ankle, hip and trunk strategies. In addition, exercises with these tools provide suitable conditions for receiving sensory information, brain decision-making, information convergence and proper nerve control during task execution. In such a way that in the process of completing the task, the patient is constantly receiving feedback and correcting the movement pattern that creates an optimal neural network (23). In general, practicing with video games is useful for reducing errors in sports. According to the evidence, while performing simulated activities with video games, due to the nature of the game that a person encounters several items, more concentration is obtained (26, 27).

Limited studies have investigated the effectiveness of exergame and the use of video games in people with ankle instability or sprains (28-33). In this regard, studies have shown that exergame and the use of video games can improve balance (28, 33). In a study conducted by Kim and his colleagues in 2019, the results showed that the static and dynamic balance measured by the Biodex balance system improved significantly in the exergame group compared to the routine treatment group (28). Shousha's study also indicated that adding exergame to common treatments can have a significant effect on static and dynamic balance (33).

But in all these studies, the postural stability has been evaluated using Biodex in the condition that the person tries to maintain the center of gravity within the range of the support level. While in athletes who have been injured, stability of postural dynamics is more important; Because these people need this type of stability more by doing sports activities. Dynamic postural stability is the ability of a person to maintain balance when transitioning from a dynamic to a static state. Dynamic activities such as jumping-landing can be a more appropriate challenge for evaluating the neuromuscular system than static tests (34). In other words, static tests do not sufficiently challenge the neuromuscular system in recreating sports activities or even daily activities, and sometimes they may not be able to show the lack of postural stability due to ease (35, 36). On the other hand, movements such as jumping-landing are more similar to sports activities, and most ankle injuries occur during these types of movements (35, 37). In fact, landing from a jump is one of the common tasks in physical activities that require dynamic stability and is also a common mechanism of ankle inversion injury (38). Thus, much attention has been paid to jump landing mechanics in ankle instability studies. Landing from a jump, which imposes large and rapid shock loads on the ankle complex, is a common dynamic maneuver that has been reported to induce a lateral ankle sprain mechanism (39). Studies show that in jump-landing, people with ankle instability have disorders in the kinetics and kinematics of movement and neuromuscular control compared to healthy people (40-42). In this way, the change in the strategies adopted by these people compared to healthy people during jump-landing can be related to the risk of re-injury. Therefore, improving these strategies and having proper stability in jumping-landing can be an effective step for the rehabilitation program of these people.

To measure dynamic postural control, first Riemann et al. used the "multiple single jump" test and checked the error score (43). But the newer method is an alternative to using the stability index and the time to stabilization as two suitable criteria for measuring dynamic pastural stability. Both indicators determine how balance is maintained when transitioning from a dynamic to a static state. These are the functional variables of neuromuscular control that can be extracted from the jump-landing movement. In the jump-landing movement, the time it takes for a person to reach the necessary stability is a good indicator that can determine the presence of defects in the stability of the postural dynamics in people with ankle instability compared to healthy people (39, 44-47). . These studies showed that people with ankle instability needed more time to restore balance in landing tasks and had more errors in postural control (39, 47). In fact, the time to stabilization is the time when the person's ground reaction force, both in the internal and external direction and in the anterior-posterior direction, reaches the limit of the stationary state, and the shorter this time is, it is considered as a positive characteristic. be (2). The stability index, like the time to stabilization, shows how far a person can disperse the ground reaction forces resulting from landing, and is actually a suitable assessment of the person's movement control (39). A 2019 systematic review has shown that people with chronic ankle instability have greater scores on the Dynamic Postural Stability Index and a longer time to stability compared to healthy people. This study reported a medium to large effect size (0.45-4.57) for these indicators (48). The stability index has higher repeatability (ICC: 0.96) and more accuracy (Standard Error of Measure: 0.03) than the time to stabilization (36). Therefore, the use of both indices can provide complete information about the stability of pastural dynamics.

Therefore, in short, from a therapeutic point of view, it is a suitable treatment that can improve the strategies adopted in the jump-landing movement and increase the postural stability. However, despite the importance of postural dynamic stability during the jump-landing movement and the increasing use of exergame in sports communities as a part of rehabilitation treatment, a study aimed at investigating the effectiveness of this therapeutic intervention on the mentioned parameters in people with chronic ankle instability It's not done. Therefore, the main question of the current study is whether exergame, compared to traditional balance exercises, can increase the stability of postural dynamics in jumping-landing movement in athletes who suffer from ankle instability, which is a challenging and functional movement for this group of people. Is it more effective?

**2. The novelty**

Overall, the evidence shows that chronic ankle instability is strongly associated with poor postural control. Therefore, improving posture control as one of the causes that predispose a person to frequent sprains and increasing injuries is of interest to researchers. One of the treatments that has received attention in the last decade is exergame. Limited studies have investigated the effectiveness of exergame and the use of video games in people with ankle instability or sprains. In this regard, two studies have shown that exergame and the use of video games can improve balance. But in these studies, the postural stability has been evaluated using Biodex in the condition that the person tries to maintain the center of gravity within the range of the support level. While in athletes who have been injured, stability of postural dynamics is more important. Dynamic postural stability is the ability of a person to maintain balance when transitioning from a dynamic to a static state. Dynamic activities such as jumping-landing can be a more appropriate challenge to evaluate the neuromuscular system than static tests. In other words, static tests do not sufficiently challenge the neuromuscular system in recreating sports activities or even daily activities, and sometimes they may not be able to reveal the lack of postural stability due to ease. On the other hand, movements such as jumping-landing are more similar to sports activities and most ankle injuries occur during these types of movements. In this regard, the results of the studies indicate that people with ankle instability have disorders in the kinetics and kinematics of movement and neuromuscular control in jumping-landing compared to healthy people. In this way, the change in the strategies adopted by these people compared to healthy people during jump-landing can be related to the risk of re-injury. Therefore, from a therapeutic point of view, it is a suitable treatment that can improve the strategies adopted in the jump-landing movement and increase the postural stability. However, despite the importance of postural dynamic stability during the jump-landing movement and the increasing use of exergame in sports communities as a part of rehabilitation treatment, a study aimed at investigating the effectiveness of this therapeutic intervention on the mentioned parameters in people with chronic ankle instability It's not done.

**3. Previous studies**

- The effect of exergame on dynamic postural stability in jump-landing movement in people with chronic ankle instability.
- So far, no study has been done in this field.
- The effect of exergame on other variables in people with chronic ankle instability
- In 2021, in Mohammadi's study, participants with at least one history of ankle sprain were treated with routine exercises or wii fit for 4 weeks. After the end of the intervention, a significant improvement in people's performance was seen in side hop test and figure of eight test (31).
- In 2021, in another study by Mohammadi, 25 people with chronic ankle instability and 25 healthy people were divided into two video game intervention groups and a control group. After 12 sessions of balance and strengthening exercises in virtual reality, people in this group showed better performance in the simple and selective reaction time test than the control group (32).
- In 2021, in the study of Shousha and his colleagues, 90 people with ankle instability were divided into 3 groups. One common treatment protocol group performed a common treatment protocol with balance exercises with Biodex and the other group performed a common treatment protocol with virtual reality exercises for 3 months. Both groups of balance exercises with virtual reality and balance exercises with Biodex system after 3 months showed a significant improvement in balance indicators in four directions and Cumberland scale. The virtual reality group showed more improvement in the anterior and posterior balance index, which was attributed to the increase in the strength ratio of dorsiflexor to plantarflexor muscles in the virtual reality group (33).
- A study was conducted in 2019 by Ki-Jong Kim with the aim of investigating the effect of therapeutic exercise using video games and comparing it with traditional treatments on balance in people with functional ankle instability. In this study, 21 people with symptoms of functional instability were included in the traditional treatment and video game group. The intervention group performed strength and balance exercises for ten minutes using Nintendo Wii Fit Plus. In the control group, people performed four ankle strength exercises using Theraband and balance exercises for ten minutes. Static and dynamic balance was measured in general, anterior-posterior and medial-lateral directions. Static balance in video game training was significantly better overall than traditional training. Dynamic balance in video game training was significantly better than traditional training at level 2, level 4 and level 8 in the medial-lateral direction. This study has shown that in patients with chronic ankle instability, training with a video game is more effective than the traditional method on balance in the general direction (static) and medial-lateral direction (dynamic) (28).
- In a pilot study in 2018 by Ki-Jong Kim, the effect of virtual reality-based exercises compared to traditional exercises on ankle functional instability was investigated. The type of randomized controlled study was one blind and the number of participants was 10 for each group. The virtual reality exercises were performed with the Nintendo Wii Fit Plus, while the traditional exercises were performed with a series of exercises with the Theraband. The change in muscle strength of the two groups and the difference between before and after the intervention were compared for each group. The virtual reality group had less improvement in muscle strength of all ankle movements than the traditional exercise group. The virtual reality group had greater improvements in plantar flexion muscle strength than other movements, while the traditional group had improvements in muscle strength in all ankle movements. Therefore, virtual reality training can be added as an optional program to the regular training program (49).
- In another study conducted by Punt and his colleagues in 2017, the effect of virtual training on walking speed, number of steps per minute, stride length, weight bearing time on one leg, weight bearing time on both legs, maximum dorsiflexion and plantar flexion And the injury rate was re-examined. 90 patients with chronic ankle instability were divided into 3 groups of wii fit exercises, common physiotherapy and control group without exercise. After 6 weeks, improvement in walking speed was observed in all 3 groups, but no significant difference was observed between the 3 groups in the temporal-spatial parameters of walking (35).
- Another study was conducted in 2016 by Punt. The aim of this study was to compare the effectiveness of exercise training using Wii Fit in patients with ankle sprains: (a) with physical therapy and (b) a control group that did not receive any treatment. 90 patients with lateral ankle sprain were randomly assigned to a Wii Fit, physical therapy or control group. Before starting the treatment and 6 weeks later, foot and ankle ability variables, pain while resting and walking, delay before returning to sports, patient satisfaction, and treatment effectiveness were measured. Six weeks after baseline measurements, foot and ankle ability scores had improved in all groups, and pain during walking had decreased. No between-group differences were observed between the Wii Fit treatment and either of the other groups. As a result, Wii Fit can be used as an exercise therapy to treat patients with ankle sprains. However, Wii Fit was no more effective than physical therapy or no exercise therapy. Patients who received no treatment showed similar results to those who received any type of exercise therapy (30).
- In 2015, Kim and colleagues investigated the effect of virtual reality-based exercises on proprioception and stability of the ankle joint in 20 patients with chronic ankle instability. In this study, people were divided into two groups: strengthening exercises and balance exercises with video games. The group of strengthening exercises plays games related to strengthening exercises 3 times a week (4 weeks) and each session for 20 minutes, and the group of balance exercises also played games related to balance exercises with the Nintendo Wii Fit Plus device. The sense of joint proprioception was compared with the Biodex isokinetic device and the sense of joint instability was compared with the Cumberland Ankle Instability Questionnaire in two groups before and after treatment. In both groups, after 4 weeks of exercises, the sense of instability decreased, but only in the group of balance exercises, the joint proprioception increased (50).

**4. Research questions**

• Does the stability index in the anteroposterior, mediolateral , and vertical directions in lateral jump-landing in the treatment groups (intervention and control) differ between each other before and after the intervention?

• Does the stability index in the anteroposterior, mediolateral , and vertical directions and the result in lateral jump-landing differ between the treatment groups (intervention and control) after the intervention?

• Is the time to stabilization in the anteroposterior, mediolateral , and vertical in lateral jump-landing in the treatment groups (intervention and control) different between before and after the intervention?

• Is the time to stabilization in the anteroposterior, mediolateral , and vertical in lateral jump-landing different between the treatment groups (intervention and control) after the intervention?

• Is there a difference between the distance covered in the one leg jump test in the treatment groups (intervention and control) between before and after the intervention?

• Is the distance covered in the one leg jump test different between the treatment groups (intervention and control) after the intervention?

• Is the time spent in the lateral jump test in the treatment groups (intervention and control) different between before and after the intervention?

• Is the time spent in the lateral jump test different between treatment groups (intervention and control) after the intervention?

• Does the level of fear of movement in treatment groups (intervention and control) differ between before and after the intervention?

• Is the level of fear of movement different between the treatment groups (intervention and control) after the intervention?

**5. Specific goals (descriptive and analytical)**

**Descriptive objectives**

• Determining the average stability index in the anteroposterior, mediolateral, and vertical directions in lateral jump-landing in treatment groups (intervention and control) before and after the intervention

• Determining the average time to stabilization in anteroposterior, mediolateral, and vertical in lateral jump-landing in the treatment groups (intervention and control) before and after the intervention.

• Determining the average distance covered in the one leg jump test in treatment groups (intervention and control) before and after the intervention

• Determining the average time spent in the lateral jump test in treatment groups (intervention and control) before and after the intervention

• Determining the average level of fear of movement in treatment groups (intervention and control) before and after the intervention

**Analytical objectives**

• Comparison of the average stability index in the anteroposterior, mediolateral, and vertical in lateral jump-landing in treatment groups (intervention and control) between before and after the intervention

• Comparison of the average stability index in the anteroposterior, mediolateral, and vertical in lateral jump-landing between the treatment groups (intervention and control) after the intervention

• Comparison of the average time to stabilization in the anteroposterior, mediolateral, and vertical in lateral jump-landing in treatment groups (intervention and control) between before and after the intervention

• Comparison of the average time to stabilization in the anteroposterior, mediolateral, and vertical in lateral jump-landing between the treatment groups (intervention and control) after the intervention

• Comparison of the average distance covered in the one leg jump test in the treatment groups (intervention and control) between before and after the intervention.

• Comparison of the distance traveled in the one leg jump test between the treatment groups (intervention and control) after the intervention

• Comparison of the time spent in the lateral jump test in treatment groups (intervention and control) between before and after the intervention

• Comparison of the time spent in the lateral jump test between treatment groups (intervention and control) after the intervention

• Comparison of the average level of fear of movement in treatment groups (intervention and control) between before and after the intervention

• Comparison of the average level of fear of movement between treatment groups (intervention and control) after the intervention

**6. Practical purposes**

If exergame can bring better results, this method can be suggested for use in clinics and sports environments, because this method is very fun and enjoyable compared to traditional exercises, and it is possible to follow it for people in different conditions and It is possible even without the supervision of a therapist. In other words, if the exergame gets good results in this study, it can be used to improve dynamic postural control and use appropriate strategies in jumping-landing movement, which is one of the common tasks in sports activities and is also a common ankle injury mechanism. be placed If the treatment groups did not show much difference, it can be estimated that both treatments are effective and efficient, and therefore, according to the conditions and possibilities, both methods can be used to improve pastural control strategies.

**7. Type of study**

This research is a randomized clinical trial.

**8. Study population**

Athletes with ankle instability

**9. Sampling method**

The samples will be selected as simple non-probability sampling.

According to Mohammadi's study and the variable of time spent in lateral jump test, in order to determine the effectiveness of exergame before and after treatment, 6 people are needed in the group (α=0.05 and (Power=0.95) and also for the difference between groups After the intervention, 13 people are needed in each group (α = 0.05 and Power = 0.8 (31). Hence, 13 people are estimated in each group.

**10. Inclusion criteria**

- Age range 18-40 years
- Both sexes
- Athlete (volleyball player, basketball player or soccer player) with moderate to intense physical activity (at least 3 times a week and more than 30 minutes each time) (53)
- Having a history of unilateral ankle sprain, at least 12 months before entering the study (more than 3 months have passed since the last sprain at the time of study entry)
- Experiencing frequent episodes of emptying the ankle or feeling unstable and having symptoms such as weakness, decreased performance during the last year (54)
- Having a score of 24 or lower on the Cumberland Ankle Instability Questionnaire.

Description: This questionnaire consists of 9 questions, the total score of which is 30, and the higher score indicates the presence of high stability in the ankle. The Persian version of the Cumberland Ankle Instability Questionnaire can be used as a reliable tool to diagnose instability and measure changes caused by therapeutic interventions in athletes with functional ankle instability (55).

- No history of surgery or fracture in the ankle or lower limb based on the patient's self-report
- Do not have a history of training with video games.

**11. Exclusion criteria**

- Do not have the ability to understand or perform the required maneuvers
- Have pain during tests
- Those participants who do not want to continue cooperation for any reason
- Absence in two consecutive and three non-consecutive sessions of therapy sessions

12. Method of implementation of the plan

After receiving the code of ethics and registering the proposal in the clinical trial system, which is one of the main conditions for the implementation of the research, we will place banners to invite qualified people in three sports physiotherapy clinics to participants from among the people who voluntarily requested to participate in the project. who have met the necessary conditions to participate in the study, explanations will be given about how to conduct the research so that the participant has complete knowledge about how to perform the steps and, if he wishes, give his informed consent in the form of signing a written consent form that is approved The Ethics Committee of Tehran University of Medical Sciences has been appointed to announce. In case of absence in two treatment sessions in a row or personal unwillingness of people at any stage of the plan, people will be removed from the group. Before the start of the project, the inclusion criteria in the form of a written questionnaire are provided to the people and if they meet all the conditions, the people will enter the research project. People are randomly placed in two intervention and control groups. Treatments are performed for 12 sessions (4 weeks and 3 sessions each week). In general, in this study, evaluations are done before and after the completion of the treatment.

**12.1. Assessments**

Evaluations are done before and after the end of the treatment period. The evaluations include 1 laboratory test, 2 clinical tests and completion of a questionnaire.

**12.1.1. Laboratory test**

In this test, a force plate device (Bertec Corporation, Columbus, OH, USA) with a sampling frequency of 500 Hz is used. In order to measure dynamic postural stability, the lateral jump-landing test is used. From this test, the stability index and the time to stabilization in different directions are extracted.

First, in order to determine the maximum horizontal jump, the participant is asked to do 3 lateral jumps with the maximum possible distance. The maximum distance traveled is considered as the maximum amount of horizontal jump. Then, because it is safe for the person, 75% of it is considered for jumping on the force plate. In other words, each person stands on two feet at a distance of 75% of the maximum horizontal jump from the center of the force plane, while the head is facing forward and the hands are placed on the hip joints, then he lands on the leg that is unstable. The participant is asked to try to regain his stability as quickly as possible after landing.

It should be noted that before the main test, a number of jumps are performed as a practice. Also, if the participant is unable to maintain his balance while landing from the jump, or his opposite leg interferes with the jump or landing, or his landing is accompanied by an additional small jump, or there is a lot of swing in his arms, trunk, and opposite leg cause the tested leg to lift from the force plane, that test is removed and repeated again. The raw data obtained from 3 successful jumps, including the changes in ground reaction force in (x, y, z) directions, are recorded on the force plane for 15 seconds after landing. The interval between each repetition is 1 minute.

**12.1.1.1. Stability index:**

- The index of stability in the internal-external and anterior-posterior directions is obtained by measuring the deviation of the X and Y components of the ground reaction force from the zero point, respectively, according to the following equations (equations 1 and 2). Also, the stability index in the vertical direction is obtained by measuring the deviation of the Z component of the ground reaction force from the person's weight (formula 3). Dynamic postural stability index is a combination of postural stability indices in internal-external, anterior-posterior and vertical directions, which is sensitive to changes in all three directions (equation 4). As can be seen in the formulas, the values ​​are normalized based on the weight of each person to make comparison between people possible. Then the average of 3 repetitions is considered as the final data (56). It should be noted that from the moment of landing (the moment when the vertical component of the ground reaction force exceeds 5% of the body weight) to 3 seconds after that is used for calculations because this time is the best choice to imitate sports performance as much as possible (36, 56).

$$\mathbf{Equation} \mathbf{1}\mathbf{:}\mathbf{MLSI}=\sqrt{\left( \frac{\sum{(0-GRFx)}^{2}}{number of data points} \right)} \div BW$$

$$\mathbf{Equation 2: APSI=}\sqrt{\left( \frac{\sum{(0-GRFy)}^{2}}{number of data points} \right)} \div BW$$

$$\mathbf{Equation 3: VSI=}\sqrt{\left( \frac{\sum{(BW-GRFz)}^{2}}{number of data points} \right)} \div BW$$

$$\mathbf{Equation 4:} \mathbf{DPSI}\boldsymbol{=}\sqrt{\left( \frac{\sum{(0-GRFx)}^{2}+ \sum{(0-GRFy)}^{2}+\sum{(BW-GRFz)}^{2}}{number of data points} \right)} \div BW$$

**12.1.1.2. Time to stabilization**

To calculate the time to stabilization, first a time series is obtained by averaging the normalized reaction forces in internal-external, anterior-posterior and vertical directions. In this method, 1 data of the reaction force is successively added to the previous data and averaging is done. As a result, a time series is obtained from the successive averaging of the reaction forces. In addition, the standard deviation and average time series of the normalized reaction forces in internal-external, anterior-posterior directions in the first 3 seconds after landing are calculated and "standard deviation ± 0.25 of the mean" is considered as a threshold. When the time series of successive averaging of the reaction forces is in the range between the upper and lower limits of the threshold, it means stability. Therefore, the moment when the time series is in this range with successive averaging is considered the moment of stability and the distance between this moment and the moment of landing is defined as the time to stabilization. For the reaction forces in the vertical direction, the threshold is defined based on "±5% of the person's weight". The resultant time to stabilization is also calculated through equation 5. Then the average of 3 repetitions is considered as the final data for each direction. It should be mentioned that to determine the body weight, the participant is asked to stand on the force plate for 5 seconds on one leg and the average vertical reaction force during this 5 seconds of standing is considered as the person's weight (36).

$$\mathbf{Equation 5:} \mathbf{RVTTS}= \sqrt{{(MLTTS)}^{2}+{(APTTS)}^{2}}$$

**12.1. 2. Clinical test (physical performance)**

In this study, single leg jump and lateral jump tests are used. In the one leg jump test, the participant is asked to jump forward with the maximum distance he can. The distance from the position of the toes on the starting line to the end of the jump is measured with a tape measure. This test has ICC=0.96 and SEM=4.56 (51). In the lateral jump test, the participant is asked to land sideways on the injured leg at a distance of 30 cm. This process is repeated 10 times. It should be noted that one repetition includes going back and forth to the starting point. The participants are asked to do this as fast as possible and the elapsed time is measured by a stopwatch. This test has ICC=0.84 and SEM=2.10 (51).

**12.1.3. Fear of movement**

In this study, Tempa questionnaire is used to check the fear of movement. This questionnaire contains eleven items, 4 options are considered for each question, and the score of each option is completely disagree = 1, disagree = 2, agree = 3 and completely agree = 4. At the end, the person can score between 11 and 44 in this questionnaire and the amount of fear of movement is measured. This test has ICC = 0.81 and SEM = 2.54 (52, 57).

**12.2. Treatments**

After completing the initial assessment, people will be randomly divided into two groups, which will be done using the randomization sequence through the randomization.com website and the balanced block randomization method. The size of the blocks is 4. Also, for the purpose of allocation concealment, sealed and numbered envelopes are used, which are provided to the participants by the secretary. The treatment period in both groups is 12 sessions and 3 times a week. Evaluations are done before and after treatment.

The participants are not blind, and according to the design of the study, it is not possible for the therapists to be blind, and the assessor becomes blind.

**12.2.1. Intervention group (exergame)**

In this group, the participant stands on the wii balance board (Nintendo Co. Ltd., Kyoto, Japan) and maintains his balance by transferring weight during various exercises. In this study, Single Leg Extension, Torso Twist, Single Leg Twist, Sideways Leg Lift, Rowing Squat, Table Tilt, Penguin Fishing, Soccer Heading, Tightrope Walk, and Snowboard Slalom games are used. In each session, each game is played for approximately 6 minutes, which makes a total of 1 hour. The course of treatment is 12 sessions (3 sessions per week). In general, each game has 3 difficulty levels (beginner, advanced and expert), and each level has 4 sub-levels (unstable, amateur, professional and champion). A person's level is determined based on the number of stars: 1 star (unstable), 2 stars (amateur), 3 stars (professional) and 4 stars (heroic). If the participant has the ability to play the game of one level, he will be upgraded to a higher level. Also, the selection of games in each session is based on the level of interest of the participants (23, 61).

**12.2.2. Control group**

The course of treatment is 12 sessions (3 times a week) and each session is 1 hour. The table below shows the progress of the treatment (10, 58-60).

The description of balance training

|  | **Difficulty Levels** | **Instructions** | **Criteria for Progression** | **Errors** |
| --- | --- | --- | --- | --- |
| **Single-Limb Stance** | 1) Eyes open, hard surface, 30 s, 3 Reps  2) Eyes open, hard surface, 60 s, 3 Reps  3) Eyes open, foam surface, 30 s, 3 Reps  4) Eyes open, foam surface, 60 s, 3 Reps  5) Eyes open, foam surface, 90 s, 3 Reps  6) Eyes open, foam surface, 30 s, ball toss, 3 Reps  7) Eyes open, foam surface, 60 s, ball toss, 3 Reps  8) Eyes open, foam surface, 90 s, ball toss, 3 Reps  9) Eyes closed, hard surface, 30 s, arms out, 3 Reps  10) Eyes closed, hard surface, 30 s, arms across, 3 Reps  11) Eyes closed, foam surface, 30 s, arms out, 3 Reps  12) Eyes closed, foam surface, 30 s, arms across, 3 Reps | The participants attempt to maintain balance on the affected limb while facing challenges such as altering the base of support, closing eyes, extending the duration, and throwing a ball [33, 34]. | If the participants can complete 3 repetitions without errors at each level of difficulty. | Errors are defined as:  A. Touching the ground with the opposite foot.  B. Excessive trunk motion (more than 30 degrees of lateral flexion).  C. Resting the opposite limb against the stance limb.  D. Lifting the hands from the chest while standing. |
| **Limb Stance with Ball Kicking** | 1) Double-limb stance, hard surface, ball Kicking, 3 Reps  2) Single- limb stance, hard surface, ball Kicking, 3 Reps  3) Double-limb stance, mini-trampoline, ball Kicking, 3 Reps  4) Single- limb stance, mini-trampoline, ball Kicking, 3 Reps | The participants are instructed to return the ball to the therapist and maintain balance as much as possible after kicking. During this training, the uninjured limb is used for kicking, while the affected limb served as the supporting limb [35]. | If the participants successfully complete 3 repetitions without any errors at each difficulty level. | Errors included A, B, C, and D mentioned earlier. |
| **Single-Limb Hop to Stabilization** | 1) Target at 18 inch, using arms to aid in stabilizing, 10 Reps  2) Target at 18 inch, hands on hips while stabilizing, 10 Reps  3) Target at 27 inch, using arms to aid in stabilizing, 10 Reps  4) Target at 27 inch, hands on hips while stabilizing, 10 Reps  5) Target at 36 inch, using arms to aid in stabilizing, 10 Reps  6) Target at 36 inch, hands on hips while stabilizing, 10 Reps | The participants are instructed to perform 10 hops in four directions:  1. Anterior-posterior  2. Medial-lateral  3. Anteromedial-posterolateral  4. Anterolateral-posteromedial  Three target distances (18, 27, or 36 inches) from the starting point are set. The participants hop to these targets, stabilize their balance on one limb, then hop back in the opposite direction to the starting position and stabilize again on one limb [33]. | If the participants successfully complete 10 repetitions without any errors at each level of difficulty. | Errors included A, B, C, and D mentioned earlier. |
| **Hop to Stabilization and reach** | 1) Target at 18 inch, using arms to aid in stabilizing, 5 Reps  2) Target at 18 inch, hands on hips while stabilizing, 5 Reps  3) Target at 27 inch, using arms to aid in stabilizing, 5 Reps  4) Target at 27 inch, hands on hips while stabilizing, 5 Reps  5) Target at 36 inch, using arms to aid in stabilizing, 5 Reps  6) Target at 36 inch, hands on hips while stabilizing, 5 Reps | The participants hop, stabilize, and reache back to the starting position. Then, they return to the starting position and reach to the target position [33]. | If the participants successfully complete 5 repetitions without errors at each difficulty level. | Errors included A, B, C, and D mentioned earlier. |

Reps= Repetitions

**13. Data collection tools**

• Individual information by way of interview and registration in the questionnaire

• Weight of samples by scale

• Length of samples by tape measure

• Checking the performance by one leg jump and side jump tests and measurement by tape measure and stopwatch

• Investigating the stability index and the time to stabilization in the jump-landing movement using the power plate device (Bertec Corporation, Columbus, OH, USA)

• Fear of movement is checked by Tempa questionnaire.

**14. Data analysis method**

First, the normal distribution of the data is done by Shapiro Wilk test. In case of normal data distribution, 2 way mixed ANOVA is used. Independent variables are group with two levels (intervention and control) and time with two levels (before and after). If there is no normal distribution, the non-parametric equation is used. The Mann-Whitney U test is used to check the effect of the group and the Wilcoxon signed-rank test is used to check the time.

**15. Ethical considerations**

1. After obtaining approval from the ethics committee of Tehran University of Medical Sciences, all stages of evaluation and testing are carried out in the rehabilitation faculty of this university.

2. Before conducting the test, all its steps, the method and purpose of doing it are explained to the people, and the participants read and completed the informed consent form.

3. The tools used in this research are non-invasive and no harm is done to the participants.

4. People participating in this study are assured that they will not be charged any fees and all the tests will be performed completely free of charge; And in case of any possible expenses due to participating in this study, the researcher will be obliged to compensate it.

5. The tests are such that they will not cause any increase in the severity of the disorder or clinical symptoms in the participants, and in case of a possible increase in the disorder, the tests will be stopped.

6. People participating in this study are allowed to withdraw from cooperation with this study at any stage of the research for any reason or without a specific reason.

7. The participants are assured that the information collected from them is protected, the principle of confidentiality is respected and the information is used only for statistical analysis.

1. Kosik KB, McCann RS, Terada M, Gribble PAJBJoSM. Therapeutic interventions for improving self-reported function in patients with chronic ankle instability: a systematic review. 2017;51(2):105-12.

2. Wright I, Neptune R, van den Bogert AJ, Nigg BJJob. The influence of foot positioning on ankle sprains. 2000;33(5):513-9.

3. Tanen L, Docherty CL, Van Der Pol B, Simon J, Schrader JJF, specialist a. Prevalence of chronic ankle instability in high school and division I athletes. 2014;7(1):37-44.

4. Hiller CE, Nightingale EJ, Lin C-WC, Coughlan GF, Caulfield B, Delahunt EJBjosm. Characteristics of people with recurrent ankle sprains: a systematic review with meta-analysis. 2011;45(8):660-72.

5. Brown CN, Padua DA, Marshall SW, Guskiewicz KMJJoat. Hip kinematics during a stop-jump task in patients with chronic ankle instability. National Athletic Trainers' Association, Inc; 2011. p. 461-7.

6. Hertel JJJoat. Functional anatomy, pathomechanics, and pathophysiology of lateral ankle instability. 2002;37(4):364.

7. Attenborough AS, Hiller CE, Smith RM, Stuelcken M, Greene A, Sinclair PJJSm. Chronic ankle instability in sporting populations. 2014;44(11):1545-56.

8. McKeon PO, Hertel JJJoat. Systematic review of postural control and lateral ankle instability, part I: can deficits be detected with instrumented testing? 2008;43(3):293-304.

9. Williams VJ, Nagai T, Sell TC, Abt JP, Rowe RS, McGrail MA, et al. Prediction of dynamic postural stability during single-leg jump landings by ankle and knee flexibility and strength. 2016;25(3):266-72.

10. Anguish B, Sandrey MAJJoAT. Two 4-week balance-training programs for chronic ankle instability. 2018;53(7):662-71.

11. Docherty CL, McLeod TCV, Shultz SJJCjosm. Postural control deficits in participants with functional ankle instability as measured by the balance error scoring system. 2006;16(3):203-8.

12. McKeon PO, Hertel JJBmd. Spatiotemporal postural control deficits are present in those with chronic ankle instability. 2008;9(1):1-6.

13. Benzing V, Schmidt MJJocm. Exergaming for children and adolescents: strengths, weaknesses, opportunities and threats. 2018;7(11):422.

14. Elena P, Demetris S, Christina M, Marios PJFiN. Differences Between Exergaming Rehabilitation and Conventional Physiotherapy on Quality of Life in Parkinson's Disease: A Systematic Review and Meta-Analysis. 2021;12:683385.

15. Adcock M, Thalmann M, Schättin A, Gennaro F, De Bruin EDJFian. A pilot study of an in-home multicomponent exergame training for older adults: feasibility, usability and pre-post evaluation. 2019;11:304.

16. Jorgensen MG, Laessoe U, Hendriksen C, Nielsen OBF, Aagaard PJJoGSABS, Sciences M. Efficacy of Nintendo Wii training on mechanical leg muscle function and postural balance in community-dwelling older adults: a randomized controlled trial. 2013;68(7):845-52.

17. Lin H, Han K, Ruan BJJoHE. Effect of Virtual Reality on Functional Ankle Instability Rehabilitation: A Systematic Review. 2021;2021.

18. de Bruin ED, Schoene D, Pichierri G, Smith STJZfGuG. Use of virtual reality technique for the training of motor control in the elderly. 2010;43(4):229-34.

19. Holden MK. Virtual environments for motor rehabilitation: review. CyberPsychol Behav 2005; 8 (3): 187–211.

20. Schultheis MT, Rizzo AAJRp. The application of virtual reality technology in rehabilitation. 2001;46(3):296.

21. Stott I, Sanders DJIJoRR. The use of virtual reality to train powered wheelchair users and test new wheelchair systems. 2000;23(4):321-6.

22. dos Santos Mendes FA, Pompeu JE, Lobo AM, da Silva KG, de Paula Oliveira T, Zomignani AP, et al. Motor learning, retention and transfer after virtual-reality-based training in Parkinson's disease–effect of motor and cognitive demands of games: a longitudinal, controlled clinical study. 2012;98(3):217-23.

23. Khanmohammadi R, Olyaei G, Talebian S, Hadian MR, Hossein B, Aliabadi SJD, et al. The effect of video game-based training on postural control during gait initiation in community-dwelling older adults: a randomized controlled trial. 2021:1-8.

24. Valentina M, Ana Š, Valentina M, Martina Š, Željka K, Mateja ZJACC. Virtual reality in rehabilitation and therapy. 2013;52(4.):453-7.

25. Gioftsidou A, Vernadakis N, Malliou P, Batzios S, Sofokleous P, Antoniou P, et al. Typical balance exercises or exergames for balance improvement? 2013;26(3):299-305.

26. Kim A, Darakjian N, Finley JM. Walking in fully immersive virtual environments: an evaluation of potential adverse effects in older adults and individuals with Parkinson's disease. J Neuroeng Rehabil. 2017;14(1):16.

27. Satyen L, Ohtsuka K. Strategies to Develop Dual Attention Skills Through Video Game Training: Ashgate; 2001.

28. Kim K-J, Heo MJJoB, Rehabilitation M. Comparison of virtual reality exercise versus conventional exercise on balance in patients with functional ankle instability: a randomized controlled trial. 2019;32(6):905-11.

29. Kim K-J, Jun H-J, Heo MJJopts. Effects of Nintendo Wii Fit Plus training on ankle strength with functional ankle instability. 2015;27(11):3381-5.

30. Punt I, Ziltener JL, Monnin D, Allet LJSjom, sports si. W ii F it™ exercise therapy for the rehabilitation of ankle sprains: Its effect compared with physical therapy or no functional exercises at all. 2016;26(7):816-23.

31. Mohammadi N, Hadian M-R, Olyaei G-RJCR. Comparison of the effects of Wii and conventional training on functional abilities and neurocognitive function in basketball-players with functional ankle instability: Matched randomized clinical trial. 2021;35(10):1454-64.

32. Mohammadi N, Hadian M-R, Olyaei G-RJJoBP, Engineering. The Effect of Wii Training on Neurocognitive Function in Athletes with Functional Ankle Instability: Matched Randomized Clinical Trial. 2021.

33. Shousha TM, Abo-zaid NA, Hamada HA, Abdelsamee MYA, Behiry MAJDoBFoPT. Virtual reality versus Biodex training in adolescents with chronic ankle instability: a randomized controlled trial. 2021.

34. Head PL, Kasser R, Appling S, Cappaert T, Singhal K, Zucker-Levin AJPTiS. Anterior cruciate ligament reconstruction and dynamic stability at time of release for return to sport. 2019;38:80-6.

35. Webster KA, Gribble PAJJoat. Time to stabilization of anterior cruciate ligament–reconstructed versus healthy knees in National Collegiate Athletic Association Division I female athletes. 2010;45(6):580-5.

36. Wikstrom EA, Tillman MD, Smith AN, Borsa PAJJoat. A new force-plate technology measure of dynamic postural stability: the dynamic postural stability index. 2005;40(4):305.

37. Krupenevich RL, Pruziner AL, Miller RHJM, sports si, exercise. Knee Joint Loading during Single-Leg Forward Hopping. 2017;49(2):327-32.

38. Shiravi Z, Shadmehr A, Moghadam ST, Moghadam BAJM, ligaments, journal t. Comparison of dynamic postural stability scores between athletes with and without chronic ankle instability during lateral jump landing. 2017;7(1):119.

39. Wikstrom EA, Tillman MD, Borsa PAJM, sports si, exercise. Detection of dynamic stability deficits in subjects with functional ankle instability. 2005;37(2):169-75.

40. Lin J-Z, Lin Y-A, Tai W-H, Chen C-YJB. Influence of Landing in Neuromuscular Control and Ground Reaction Force with Ankle Instability: A Narrative Review. 2022;9(2):68.

41. Moisan G, Mainville C, Descarreaux M, Cantin VJJoAT. Lower limb biomechanics during drop jump landing in individuals with chronic ankle instability. 2022.

42. Kim H, Son SJ, Seeley MK, Hopkins JTJSjom, sports si. Altered movement strategies during jump landing/cutting in patients with chronic ankle instability. 2019;29(8):1130-40.

43. Riemann BL, Caggiano NAJJoSR. Examination of a Clinical Method of Assessing Postural Control During a Functional. 1999;8:171-83.

44. Ross SE, Guskiewicz KMJIJoAT, Training. Time to stabilization: a method for analyzing dynamic postural stability. 2003;8(3):37-9.

45. Brown CN, Mynark RJJoat. Balance deficits in recreational athletes with chronic ankle instability. 2007;42(3):367.

46. Ross SE, Guskiewicz KM, Gross MT, Yu BJJoat. Assessment tools for identifying functional limitations associated with functional ankle instability. 2008;43(1):44-50.

47. Ross SE, Guskiewicz KM, Yu B. Single-leg jump-landing stabilization times in subjects with functionally unstable ankles. Journal of athletic training. 2005;40(4):298.

48. Simpson JD, Stewart EM, Macias DM, Chander H, Knight ACJPTiS. Individuals with chronic ankle instability exhibit dynamic postural stability deficits and altered unilateral landing biomechanics: A systematic review. 2019;37:210-9.

49. Kim K, Choi B, Lim WJD, Technology RA. The efficacy of virtual reality assisted versus traditional rehabilitation intervention on individuals with functional ankle instability: a pilot randomized controlled trial. 2019;14(3):276-80.

50. Kim KJJJoIAoPTR. Effects of virtual reality programs on proprioception and instability of functional ankle instability. 2015;6(2):891-5.

51. Sharma N, Sharma A, Sandhu JSJAjosm. Functional performance testing in athletes with functional ankle instability. 2011;2(4):249.

52. Woby SR, Roach NK, Urmston M, Watson PJJP. Psychometric properties of the TSK-11: a shortened version of the Tampa Scale for Kinesiophobia. 2005;117(1-2):137-44.

53. Lynall RC, Campbell KR, Mauntel TC, Blackburn JT, Mihalik JPJJoat. Single-legged hop and single-legged squat balance performance in recreational athletes with a history of concussion. 2020;55(5):488-93.

54. Gribble PA, Delahunt E, Bleakley C, Caulfield B, Docherty C, Fourchet F, et al. Selection criteria for patients with chronic ankle instability in controlled research: a position statement of the International Ankle Consortium. 2013;43(8):585-91.

55. Mirshahi M, Halabchi F, Golbakhsh M, Saadat SJAJoEM. Reliability and recalibration of the Persian version of Cumberland Ankle Instability Tool cut-off score in athletes with functional ankle instability. 2019;3(3).

56. Rawcliffe AJ, Hinde KL, Graham SM, Martindale R, Morrison A, Krajewski KT, et al. Altered dynamic postural stability and joint position sense following British Army foot-drill. 2020;2.

57. Rahmati N, Moghadam MAA, Shairi MR, Paknejad M, Rahmati Z, Ghassami M, et al. Psychometric properties of the tampa scale for kinesiophobia amongst iranian patients with chronic persistent pain. 2014;13(2):197-210.

58. Conceição JS, Schaefer de Araújo FG, Santos GM, Keighley J, Dos Santos MJJJoat. Changes in postural control after a ball-kicking balance exercise in individuals with chronic ankle instability. 2016;51(6):480-90.

59. McKeon PO, Ingersoll CD, Kerrigan DC, Saliba E, Bennett BC, Hertel JJM, et al. Balance training improves function and postural control in those with chronic ankle instability. 2008;40(10):1810-9.

60. Hale SA, Hertel J, Olmsted-Kramer LCJJoo, therapy sp. The effect of a 4-week comprehensive rehabilitation program on postural control and lower extremity function in individuals with chronic ankle instability. 2007;37(6):303-11.

61. Bagheri H, Khanmohammadi R, Olyaei G, Talebian S, Hadian MR, Najafi MJNL. Video game and motor-cognitive dual-task training could be suitable treatments to improve dual-task interference in older adults. 2021;760:136099.
