# Supplementary material for: Comparison of the effects of exergaming and balance training on dynamic postural stability during jump-landing in recreational athletes with chronic ankle instability
Source: PLoS One. 2024 Dec 16;19(12):e0314686. doi: 10.1371/journal.pone.0314686 (PMC11649137; doi:10.1371/journal.pone.0314686)
Supplement: S2 Text — (DOCX) [file pone.0314686.s003.docx]

**مقایسه اثرات exergame و تمرینات تعادلی بر ثبات پاسچرال داینامیک در حرکت پرش- فرود در ورزشکاران مبتلا به بی ثباتی مزمن مچ پا**

صدف سپاسگزار، رویا خانمحمدی، زینب شیروی

**1. مقدمه و بیان مسئله**

مچ پا شایع ترین مفصل آسیب دیده در آسیب های ورزشی است بگونه ای که 10 تا 30 درصد از آسیب های ورزشی را شامل می شود (1). طبق شواهد و مطالعات انجام گرفته پیچ خوردگی مچ پا^[[1]](#footnote-2)^ یک عارضه همیشگی با اثرات طولانی مدت بوده و می تواند تاثیرات مخربی بر عملکرد فرد ورزشکار داشته باشد. پیچ خوردگی مچ پا یک ششم کل زمان از دست رفته از شرکت در مسابقات ورزشی را تشکیل می دهد (2). بعبارتی پیچ خوردگی مچ پا علاوه بر خسارات مالی زیاد سبب دوری افراد از فعالیتهای ورزشی می شود. تقریباً 33 درصد از افرادی که پیچ‌خوردگی جانبی مچ پا را تجربه میکنند دچار بی ‌ثباتی مزمن مچ پا^[[2]](#footnote-3)^ می‌ شوند (3, 4). بی ثباتی مزمن به تکرار خالی کردن مچ پا^[[3]](#footnote-4)^ بعد از پیچ خوردگی اولیه و نقص در کنترل عصبی عضلانی و ثبات مکانیکی گفته می شود (5, 6). درصد بالایی (%61 -%23) از ورزشکاران، دارای بی ثباتی مزمن مچ پا هستند که فوتبال، بسکتبال و والیبال بیشترین تیم های ورزشی معرفی شده در این آسیب به شمار میآیند (7).

در اواسط سال 1960، Freeman اینگونه مطرح کرد که بی ثباتی مزمن مچ پا در ارتباط قوی با کنترل پاسچرال ضعیف است (8). کنترل پاسچرال توانایی حفظ تعادل از طریق نگه داشتن مرکز ثقل روی سطح اتکا^[[4]](#footnote-5)^ است. حفظ کنترل پاسچرال مستلزم ادغام اطلاعات حسی و اجرای پاسخ های حرکتی مناسب است (9). اما در این افراد بدنبال آسیب به عضلات و ساختارهای عصبی محیطی، مهار سیستم عصبی مرکزی و سازماندهی مجدد حسی، و اختلال عصبی حرکتی، کنترل پاسچرال و تعادل دچار اختلال میشود (10). کنترل پاسچرال برای مشارکت و عملکرد ورزشی بسیار مهم و ضروری است اما در افراد مبتلا به پیچ خوردگی و یا بی ثباتی مچ پا کاهش می یابد (11). شیوع بالای آسیب دیدگی مچ پا و ماندگاری علائم بی ثباتی تا مدتهای طولانی، ممکن است باعث بوجود آمدن اختلالاتی در مفصل آسیب دیده شود که در صورت عدم درمان مناسب میتواند در فعالیتهای ورزشی و عملکرد روزمره فرد، مشکلات قابل توجهی ایجاد کند (12). از این رو بهبود کنترل پاسچر به عنوان یکی از علل مستعد کننده فرد به پیچ خوردگی مکرر و بیشتر شدن آسیب، مورد توجه محققین میباشد.

از منظر درمانی درمانهای مختلفی برای بهبود تعادل و کنترل پاسچرال در افراد مبتلا به بی ثباتی مچ پا پیشنهاد شده است. یکی از درمانهایی که در دهه اخیر مورد توجه قرار گرفته exergame میباشد. Exergame از دو کلمه ورزش و بازی تشکیل شده و به معنای استفاده از بازیهای کامپیوتری به منظور افزایش سطح فعالیت فیزیکی میباشد (13). Exergame یک فناوری جدید است که می تواند برای بهبود تعادل مورد استفاده قرار گیرد و در حال تبدیل شدن به یک روش مداخله رایج در جهان است (13). در این روش توانبخشی، بازی های دیجیتالی به فرد ارائه می شود و کاربر، تمریناتی را برای دستیابی به نتایج بازی انجام می دهد. این بازیهای دیجیتالی میتواند بدون غوطه ور شدن فرد در فضای مجازی^[[5]](#footnote-6)^ باشد یا با نیمه غوطه ور شدن یا با غوطه وری کامل وی همراه باشد (14).

اگرچه اثرات تمرینات سنتی بر تقویت حس تعادل ثابت شده است، اما این برنامه‌ها از این جهت که تا حدودی خسته‌کننده هستند و نمی‌توانند علاقه شرکت‌کنندگان را برانگیزند، دارای نقطه ضعف هستند زیرا در برخی موارد باعث میشود فرد قبل از کسب نتایج مطلوب، دوره درمان را رها کند اما بازیهای ویدیویی جذاب و سرگرم کننده هستند (15, 16). همچنین در برنامه های تمرینی سنتی از آنجایی که افراد نمیتوانند از یک باز خورد^[[6]](#footnote-7)^ آنی برخوردار باشند برای مشارکت فعال ترغیب نمی شوند اما در بازیهایی ویدیویی مکررا به فرد فیدبکهای دیداری و شنوایی داده میشود که میزان مشارکت فرد را افزایش میدهد (17). استفاده از بازیهای کامپیوتری می تواند امکان شخصی‌سازی درمان و مستندسازی پیشرفت را فراهم سازد و باعث ایجاد انگیزه و افزایش مشارکت بیماران شود (18-20). همچنین از آن جایی که به نظارت آنچنانی نیاز ندارد ممکن است بار کاری درمانگر را کاهش دهد (21). همچنین به طور کلی به صورت تجاری در دسترس است و می تواند برای توانبخشی در منزل استفاده شود. بازی های کامپیوتری میتوانند چندین منبع شناختی و حرکتی مختلف مانند مهار کردن محرک های غیر مرتبط، تصمیم گیری، تمرکز و قدرت و تعادل را هدف قرار دهند (22). درگیر شدن منابع شناختی از مزایای مهم این برنامه توانبخشی است. مطالعات نشان داده اند که در افراد مبتلا به بی ثباتی مچ پا، بارشناختی^[[7]](#footnote-8)^ میتواند بعنوان یک عامل خطر در پیچ خوردگی های مکرر درنظر گرفته شود. درحقیقت در این دسته از افراد اختلال در پردازش اطلاعات وجود دارد. از این رو افزایش مهارت های حرکتی همزمان با کاهش وابستگی به پردازش آگاهانه ی اطلاعات و استفاده از تمرینات دوگانه و چندگانه و افزایش ظرفیت پردازش سیستم عصبی مرکزی می تواند مفید باشد. بهم پیوستگی منابع مورد نیاز حرکتی و شناختی در بسیاری از تسکها منجر به طراحی درمان هایی شده است که در آن تمرینات شناختی و حرکتی با یکدیگر ترکیب شده اند و برای بهبود کنترل پاسچر به کار می روند. در واقع محققین عقیده دارند که تمرینات ترکیبی حرکتی و شناختی اثر بیشتری نسبت به تمرینات حرکتی یا شناختی به تنهایی دارند (23). امروزه یک گزینه ی امیدوارکننده برای تمرینات تلفیقی حرکتی و شناختی، بازی های ویدیویی هستند (23). علاوه بر این، این سیستم می تواند یک محیط توانبخشی را فراهم کند که به کاربران این امکان را بدهد که به تکرار تمرینات بپردازند، در حالی که انگیزه کافی برای انجام تعداد زیادی تمرین را دارند که همه این موارد به عنوان مولفه های مهم در توانبخشی شناخته می شوند (24). عامل دیگری که باعث بهبود تعادل پس از انجام تمرینات با بازیهای ویدئویی می شود مقدار فیدبک هایی هست که کاربر حین بازی دریافت می کند. فیدبک همزمان به کاربر اجازه می دهد که روی حرکات خود تمرکز بیشتری داشته باشد. در بازیها فیدبک ها بر اساس عملکرد و نتیجه به وی داده میشود که می تواند باعث یادگیری بهتر تسک های حرکتی شود (25). از مزایای دیگر بازی های ویدیویی آن است که در حین تمرینات، کاربر باید دائما وزن خود را در جهات مختلف با سرعت و شدت های مختلف انتقال دهد در حالی که بایستی مرکز فشار را در سطح اتکای عملکردی خود حفظ کند. این حرکات کنترل شده بسیار شبیه به استراتژی های مچ پا، لگن و تنه می باشد. علاوه بر این، تمرینات با این ابزارها شرایط مناسبی برای دریافت اطلاعات حسی، تصمیم گیری مغز، همگرایی اطلاعات و کنترل مناسب اعصاب حین اجرای تسک فراهم میکند. بگونه ای که در روند کامل کردن تسک، بیمار دائما در حال دریافت فیدبک و اصلاح الگوی حرکت می باشد که یک شبکه ی عصبی بهینه را به وجود آورد (23). به طور کلی تمرین کردن با بازی های ویدیویی برای کم کردن خطاها در ورزش مفید می باشد. بنابر شواهد، حین انجام فعالیت های شبیه سازی شده با بازی های ویدیویی، به دلیل خاصیت بازی که فرد با چندین آیتم مواجه می شود تمرکز بیشتری به دست می آید (26, 27).

مطالعات محدودی اثر بخشی exergame و استفاده از بازیهای ویدیویی را در افراد مبتلا به بی ثباتی و یا پیچ خوردگی مچ پا را بررسی کرده اند (28-33). در این راستا مطالعات نشان داده اند که exergame و استفاده از بازیهای ویدیویی میتوانند تعادل را بهبود بخشند (28, 33). در مطالعه ای که Kim و همکارانش درسال 2019 انجام دادند نتایج نشان داد که تعادل استاتیک و دینامیک که به وسیله سیستم تعادلی بایودکس مورد اندازه گیری قرار گرفته بود در گروه exergame نسبت به گروه درمانهای روتین به طور قابل توجهی بهبود پیدا کرد (28). مطالعه Shousha نیز بیانگر آن بود که اضافه کردن exergame به درمانهای رایج میتواند اثر چشمگیری بر تعادل استاتیک و داینامیک داشته باشد (33).

اما در تمامی این مطالعات، ثبات پاسچرال با استفاده از بایودکس در شرایطی ارزیابی شده است که فرد تلاش میکند تا مرکز ثقل را در محدوده سطح اتکا حفظ کند. در حالیکه در افراد ورزشکاری که دچار آسیب شده اند ثبات پاسچرال داینامیک از اهمیت بیشتری برخوردار است؛ زیرا این افراد بواسطه انجام فعالیتهای ورزشی به این نوع ثبات نیاز بیشتری دارند. ثبات پاسچرال داینامیک، توانایی فرد برای حفظ تعادل هنگام انتقال از حالت داینامیک به استاتیک است. فعالیت های داینامیک همانند پرش- فرود^[[8]](#footnote-9)^ میتوانند چالش مناسب تری برای ارزیابی سیستم عصبی عضلانی نسبت به آزمون استاتیک باشند (34). بعبارتی آزمونهای استاتیک، سیستم عصبی عضلانی را در بازآفرینی فعالیت های ورزشی یا حتی فعالیت های روزمره، به طور کافی به چالش نمی کشند و گاها ممکن است به دلیل آسانی، نتوانند نقصان ثبات پاسچرال را نمایان سازند (35, 36). از سوی دیگر حرکاتی مانند پرش- فرود به فعالیتهای ورزشی تشابه بیشتری دارند و بیشتر آسیبهای مچ پا در حین این نوع حرکات اتفاق می افتد (35, 37). در حقیقت فرود آمدن از پرش یکی از کارهای رایج در فعالیت های بدنی است که به ثبات داینامیک نیاز دارد و همچنین یک مکانیسم رایج آسیب اینورژن مچ پا است (38). به این ترتیب، در مطالعات مربوط به بی ثباتی مچ پا به مکانیک فرود از پرش توجه زیادی شده است. فرود از پرش که بارهای ضربه ای بزرگ و سریع را به مجموعه مچ پا تحمیل می کند، یک مانور داینامیکی معمول گزارش شده است که مکانیسم پیچ خوردگی جانبی مچ پا را منجر می شود (39). مطالعات بیانگر آن است که در پرش- فرود افراد مبتلا به بی ثباتی مچ پا در مقایسه با افراد سالم اختلالاتی در کینتیک و کینماتیک حرکت و کنترل عصبی عضلانی دارند (40-42). به این ترتیب تغییر در استراتژیهای اتخاذ شده این افراد در مقایسه با افراد سالم طی انجام پرش- فرود میتواند در ارتباط با خطر آسیب مجدد باشد. از این رو بهبود این استراتژیها و داشتن ثبات مناسب در پرش- فرود میتواند یک گام موثر برای برنامه توانبخشی این افراد باشد.

برای اندازه گیری کنترل پاسچرال داینامیک، ابتدا Riemann و همکاران از تست "تک پرش چندگانه"^[[9]](#footnote-10)^ و بررسی نمره خطا استفاده کردند (43). اما روش جدیدتر جایگزین استفاده از شاخص ثبات^[[10]](#footnote-11)^ و زمان رسیدن به ثبات^[[11]](#footnote-12)^ به عنوان دو معیار مناسب برای سنجش ثبات پاسچرال داینامیک است. هر دو شاخص تعیین میکنند که چگونه تعادل هنگام انتقال از وضعیت داینامیک به استاتیک حفظ می شود. اینها متغیرهای فانکشنال کنترل عصبی عضلانی هستند که از حرکت پرش- فرود قابل استخراج هستند. در حرکت پرش- فرود، مدت زمانی که طول میکشد تا فرد به ثبات لازم برسد شاخص مناسبی است که میتواند وجود نقص در ثبات پاسچرال داینامیک را در افراد مبتلا به بی ثباتی مچ پا نسبت به افراد سالم مشخص سازد (39, 44-47). این مطالعات نشان دادند که افراد مبتلا به بی ثباتی مچ پا به زمان بیشتری برای بازگرداندن تعادل در وظایف فرود نیاز داشته و خطاهای بیشتری در کنترل پوسچرال دارند (39, 47). در واقع زمان رسیدن به ثبات، مدت زمانی است که نیروی عکس العمل زمین فرد چه در جهت داخلی خارجی و چه در جهت قدامی خلفی، به حد وضعیت سکون میرسند و هر چه این زمان کوتاه تر باشد به عنوان یک مشخصه مثبت در نظر گرفته می شود (2). شاخص ثبات هم همانند زمان رسیدن به ثبات نشان دهنده این است که یک فرد تا چه حد می تواند نیروهای عکس العمل زمین حاصل از فرود را پراکنده سازد و در واقع ارزیابی مناسبی از کنترل حرکتی فرد است (39). یک مطالعه مروری نظام مند در سال 2019 نشان داده است که افراد مبتلا به بی ثباتی مزمن مچ پا در مقایسه با افراد سالم نمرات بزرگتری در رابطه با شاخص ثبات پاسچرال داینامیک و زمان بیشتری در رسیدن به ثبات اخذ می کنند. این مطالعه اندازه اثر متوسط تا بزرگ (57/4-45/0) برای این شاخص ها گزارش کرده است (48). شاخص ثبات نسبت به زمان رسیدن به ثبات، از تکرارپذیری بالاتر (ICC: 0.96) و دقت بیشتری (Standard Error of Measure: 0.03) برخوردار است (36). از این رو استفاده از هر دو شاخص میتواند اطلاعات کاملی درخصوص ثبات پاسچرال داینامیک ارایه دهد.

بنابراین به طور خلاصه از منظر درمانی، درمانی مناسب است که بتواند استراتژیهای اتخاذ شده در حرکت پرش- فرود را بهبود بخشیده و باعث افزایش ثبات پاسچرال شود. اما باوجود اهمیت ثبات پاسچرال داینامیک حین انجام حرکت پرش- فرود و استفاده روز افزون از exergame در جوامع ورزشی به عنوان بخشی از درمان توانبخشی، مطالعه ای در جهت بررسی اثر بخشی این مداخله درمانی بر پارامترهای مذکور در افراد مبتلا به بی ثباتی مزمن مچ پا انجام نشده است. از این رو سوال اصلی مطالعه حاضر آن است که آیا در ورزشکارانی که مبتلا به بی ثباتی مچ پا هستند exergame در مقایسه با تمرینات تعادلی سنتی میتواند در افزایش ثبات پاسچرال داینامیک در حرکت پرش- فرود که حرکتی چالش برانگیز و عملکردی برای این دسته از افراد است موثرتر باشد؟

**2.جنبة جديد بودن موضوع**

به طور کلی شواهد نشان میدهد که بی ثباتی مزمن مچ پا در ارتباط قوی با کنترل پاسچرال ضعیف است. از این رو بهبود کنترل پاسچر به عنوان یکی از علل مستعد کننده فرد به پیچ خوردگی مکرر و بیشتر شدن آسیب، مورد توجه محققین میباشد. یکی از درمانهایی که در دهه اخیر مورد توجه قرار گرفته exergame میباشد. مطالعات محدودی اثر بخشی exergame و استفاده از بازیهای ویدیویی را در افراد مبتلا به بی ثباتی و یا پیچ خوردگی مچ پا را بررسی کرده اند. در این راستا دو مطالعه نشان داده اند که exergame و استفاده از بازیهای ویدیویی میتوانند تعادل را بهبود بخشند. اما در این مطالعات، ثبات پاسچرال با استفاده از بایودکس در شرایطی ارزیابی شده است که فرد تلاش میکند تا مرکز ثقل را در محدوده سطح اتکا حفظ کند. در حالیکه در افراد ورزشکاری که دچار آسیب شده اند ثبات پاسچرال داینامیک از اهمیت بیشتری برخوردار است. ثبات پاسچرال داینامیک، توانایی فرد برای حفظ تعادل هنگام انتقال از حالت داینامیک به استاتیک است. فعالیت های داینامیک همانند پرش- فرود میتوانند چالش مناسب تری برای ارزیابی سیستم عصبی عضلانی نسبت به آزمون استاتیک باشند. بعبارتی آزمونهای استاتیک، سیستم عصبی عضلانی را در بازآفرینی فعالیت های ورزشی یا حتی فعالیت های روزمره، به طور کافی به چالش نمی کشند و گاها ممکن است به دلیل آسانی، نتوانند نقصان ثبات پاسچرال را نمایان سازند. از سوی دیگر حرکاتی مانند پرش- فرود به فعالیتهای ورزشی تشابه بیشتری دارند و بیشتر آسیبهای مچ پا در حین این نوع حرکات اتفاق می افتد. در این راستا نتایج مطالعات بیانگر آن است که در پرش- فرود افراد مبتلا به بی ثباتی مچ پا در مقایسه با افراد سالم اختلالاتی در کینتیک و کینماتیک حرکت و کنترل عصبی عضلانی دارند. به این ترتیب تغییر در استراتژیهای اتخاذ شده این افراد در مقایسه با افراد سالم طی انجام پرش- فرود میتواند در ارتباط با خطر آسیب مجدد باشد. از این رو از منظر درمانی، درمانی مناسب است که بتواند استراتژیهای اتخاذ شده در حرکت پرش- فرود را بهبود بخشیده و باعث افزایش ثبات پاسچرال شود. اما باوجود اهمیت ثبات پاسچرال داینامیک حین انجام حرکت پرش- فرود و استفاده روز افزون از exergame در جوامع ورزشی به عنوان بخشی از درمان توانبخشی، مطالعه ای در جهت بررسی اثر بخشی این مداخله درمانی بر پارامترهای مذکور در افراد مبتلا به بی ثباتی مزمن مچ پا انجام نشده است.

**3. سابقه علمی پژوهش های انجام شده**

- **اثرexergame بر ثبات پاسچرال داینامیک در حرکت پرش- فرود در افراد مبتلا به بی ثباتی مزمن مچ پا**

تا کنون مطالعه ای در این زمینه انجام نشده است.

- **اثرexergame بر سایر متغیرها در افراد مبتلا به بی ثباتی مزمن مچ پا**
- در سال 2021 در مطالعه ی محمدی، افراد شرکت کننده با حداقل یک سابقه ی پیچ خوردگی مچ پا به مدت 4 هفته تحت درمان با تمرینات روتین و یا wii fit قرار گرفتند. پس از پایان مداخله بهبود قابل توجه در عملکرد افراد در تست های side hop test وfigure of eight test دیده شد (31).
- در سال 2021 در مطالعه ای دیگر از محمدی 25 فرد با بی ثباتی مزمن مچ پا و 25 فرد سالم به دو گروه مداخله ی بازی های ویدیویی و گروه کنترل تقسیم شدند. پس از 12 جلسه تمرینات تعادلی و تقویتی در واقعیت مجازی افراد در این گروه نسبت به گروه کنترل عملکرد بهتری در تست زمان عکس العمل ساده و انتخابی نشان دادند (32).
- در سال 2021 در مطالعه ی Shousha و همکارانش، 90 فرد مبتلا به بی ثباتی مچ پا به 3 گروه تقسیم شدند. یک گروه پروتکل درمانی رایج، یک پروتکل درمانی رایج بهمراه تمرینات تعادلی با بایودکس و گروه دیگر پروتکل درمانی رایج بهمراه تمرینات واقعیت مجازی را به مدت 3 ماه انجام دادند. هر دو گروه تمرینات تعادلی با واقعیت مجازی و تمرینات تعادلی با سیستم بایودکس پس از 3 ماه پیشرفت چشم گیری در شاخص های تعادلی در چهارجهت و مقیاس کامبرلند^[[12]](#footnote-13)^ نشان دادند. گروه واقعیت مجازی بیشتر در شاخص تعادلی قدامی و خلفی پیشرفت نشان دادند که علت آن را به افزایش نسبت قدرت عضلات دورسی فلکسور به پلانتارفلکسور در گروه واقعیت مجازی نسبت دادند (33).
- مطالعه ای در سال 2019 توسطKi-Jong Kim با هدف بررسی تاثیر تمرین درمانی با استفاده از بازی های ویدئویی و مقایسه ی آن با درمان های سنتی بر روی تعادل در افراد با بی ثباتی عملکردی مچ پا انجام شد. در این مطالعه 21 نفر با علائم بی ثباتی عملکردی در گروه درمان سنتی و بازی ویدئویی قرار گرفتند. گروه مداخله تمرینات مبتنی بر قدرت و تعادل را برای مدت زمان ده دقیقه با استفاده از Nintendo Wii Fit Plus انجام دادند. در گروه کنترل افراد چهار تمرین قدرتی مچ پا را با استفاده از تراباند و ورزش های تعادلی برای مدت ده دقیقه انجام دادند. تعادل ایستا و پویا در جهات کلی، قدامی-خلفی و داخلی-جانبی اندازه گیری شد. تعادل ایستا در تمرین با بازی ویدیویی در جهت کلی به طور قابل توجهی بهتر از تمرین سنتی بود. تعادل پویا در تمرین با بازی ویدیویی به طور قابل توجهی بهتر از تمرین سنتی در سطح 2، سطح 4 و سطح 8 در جهت میانی - جانبی بود. این مطالعه نشان داده است که در بیماران مبتلا به بی ثباتی مزمن مچ پا تمرین با بازی ویدیویی نسبت به روش سنتی بر تعادل در جهت کلی (ایستا) و جهت داخلی-جانبی (داینامیک) موثرتر است (28).
- دریک مطالعه ی پایلوت در سال 2018 توسطKi-Jong Kim تاثیر تمرینات مبتنی بر واقعیت مجازی در مقایسه با تمرینات سنتی بر روی بی ثباتی عملکردی مچ پا بررسی شد. نوع مطالعه کنترل‌شده تصادفی یک سوکور و تعداد افراد شرکت کننده 10 نفر برای هر گروه بود. تمرینات واقعیت مجازی با Nintendo Wii Fit Plus انجام شد، در حالی که تمرینات سنتی با یک سری تمرینات با تراباند انجام گرفت. تغییر قدرت عضلانی دو گروه و تفاوت بین قبل و بعد از مداخله برای هر گروه مقایسه شد. گروه واقعیت مجازی نسبت به گروه تمرینات سنتی بهبود کمتری در قدرت عضلانی تمام حرکات مچ پا داشت. گروه واقعیت مجازی در قدرت عضلانی حرکت پلنتار فلکشن نسبت به سایر حرکات بهبود بیشتری داشت، در حالی که گروه سنتی در تمام حرکات مچ پا بهبود در قدرت عضلانی داشتند. از این رو آموزش واقعیت مجازی میتواند به عنوان یک برنامه اختیاری به برنامه تمرینی معمولی اضافه شود (49).
- در مطالعه ی دیگری کهpunt و همکارانش در سال 2017 انجام دادند اثر تمرینات مجازی بر روی سرعت راه رفتن، تعداد قدم در دقیقه، طول گام، زمان وزن اندازی روی یک پا، زمان وزن اندازی روی 2 پا، حداکثر دورسی فلکشن و پلانتار فلکشن و نرخ آسیب دوباره بررسی شد. 90 بیمار با بی ثباتی مزمن مچ پا به 3 گروه تمرینات wii fit ، فیزیوتراپی رایج و گروه کنترل بدون تمرین تقسیم شدند. پس از 6 هفته در هر 3 گروه بهبود در سرعت راه رفتن مشاهده شد ولی تفاوت معناداری بین 3 گروه در پارامترهای زمانی-فضایی راه رفتن مشاهده نشد (35).
- مطالعه ی دیگری در سال 2016 توسط Punt انجام شد. هدف از این مطالعه مقایسه اثربخشی تمرین ورزشی با استفاده از Wii Fit در بیماران مبتلا به پیچ خوردگی مچ پا بود: (الف) با فیزیوتراپی و (ب) یک گروه کنترل که هیچ درمانی دریافت نمی کرد. 90 بیمار با پیچ خوردگی جانبی مچ پا به طور تصادفی در یک گروه Wii Fit، فیزیوتراپی و یا گروه کنترل قرار گرفتند. قبل از شروع درمان و 6 هفته پس از آن متغیرهای توانایی پا و مچ پا^[[13]](#footnote-14)^، درد در هنگام استراحت و راه رفتن، تاخیر قبل از بازگشت به ورزش، رضایت بیمار، و اثربخشی درمان اندازه گیری شد. شش هفته پس از اندازه گیری های پایه، نمرات توانایی پا و مچ پا در همه گروه ها بهبود یافته بود و درد در حین راه رفتن کاهش یافته بود. هیچ تفاوت بین گروهی بین درمان Wii Fit و هر دو گروه دیگر مشاهده نشد. در نتیجه Wii Fit می تواند به عنوان یک ورزش درمانی برای درمان بیماران مبتلا به پیچ خوردگی مچ پا استفاده شود. با این حال، Wii Fit نسبت به فیزیوتراپی یا عدم انجام هرگونه ًورزش درمانی مؤثرتر نبود. بیمارانی که درمان دریافت نکردند نتایج مشابهی با افرادی که هر نوع ورزش درمانی دریافت کردند نشان دادند (30).
- در سال 2015 Kim و همکارانش اثر تمرینات مبتنی بر واقعیت مجازی را بر حس عمقی و ثبات مفصل مچ پا در 20 بیمار مبتلا به بی ثباتی مزمن مچ پا بررسی کردند. در این مطالعه افراد به دو گروه تمرینات تقویتی و تمرینات تعادلی با بازیهای ویدیویی تقسیم شدند. گروه تمرینات تقویتی 3 بار در هفته (4 هفته) و هر جلسه 20 دقیقه بازیهای مربوط به تمرینات تقویتی را انجام میداند و گروه تمرینات تعادلی نیز در همین مدت بازیهای مربوط به تمرینات تعادلی را با دستگاه نینتندو وی فیت پلاس انجام دادند. حس عمقی مفصل با دستگاه ایزوکینتیک بایودکس و حس بی ثباتی مفصل با پرسشنامه بی ثباتی مچ پا کامبرلند در دو گروه قبل و بعد از درمان مقایسه شد. در هر دو گروه پس از 4 هفته انجام تمرینات کاهش حس بی ثباتی دیده شد اما تنها در گروه تمرینات تعادلی افزایش حس عمقی مفصل دیده شد (50).

**4. سوالات پژوهش**

- آیا شاخص ثبات در جهات قدامی- خلفی/ داخلی- خارجی/ عمودی و برآیند در پرش – فرود رو به لترال در گروه های درمانی (مداخله و کنترل) بین قبل و بعد از مداخله با یکدیگر تفاوت دارد؟
- آیا شاخص ثبات در جهات قدامی- خلفی/ داخلی- خارجی/ عمودی و برآیند در پرش – فرود رو به لترال بین گروه های درمانی (مداخله و کنترل) بعد از مداخله با یکدیگر تفاوت دارد؟
- آیا زمان رسیدن به ثبات در جهات قدامی- خلفی/ داخلی- خارجی و برآیند در پرش – فرود رو به لترال در گروه های درمانی (مداخله و کنترل) بین قبل و بعد از مداخله با یکدیگر تفاوت دارد؟
- آیا زمان رسیدن به ثبات در جهات قدامی- خلفی/ داخلی- خارجی و برآیند در پرش – فرود رو به لترال بین گروه های درمانی (مداخله و کنترل) بعد از مداخله با یکدیگر تفاوت دارد؟
- آیا مسافت طی شده در تست پرش تک پا در گروه های درمانی (مداخله و کنترل) بین قبل و بعد از مداخله با یکدیگر تفاوت دارد؟
- آیا مسافت طی شده در تست پرش تک پا بین گروه های درمانی (مداخله و کنترل) بعد از مداخله با یکدیگر تفاوت دارد؟
- آیا زمان سپری شده در تست پرش جانبی در گروه های درمانی (مداخله و کنترل) بین قبل و بعد از مداخله با یکدیگر تفاوت دارد؟
- آیا زمان سپری شده در تست پرش جانبی بین گروه های درمانی (مداخله و کنترل) بعد از مداخله با یکدیگر تفاوت دارد؟
- آیا میزان ترس از حرکت در گروه های درمانی (مداخله و کنترل) بین قبل و بعد از مداخله با یکدیگر تفاوت دارد؟
- آیا میزان ترس از حرکت بین گروه های درمانی (مداخله و کنترل) بعد از مداخله با یکدیگر تفاوت دارد؟

**5. اهداف اختصاصی (توصیفی و تحلیلی)**

**اهداف توصیفی**

- تعیین میانگین شاخص ثبات در جهات قدامی- خلفی/ داخلی- خارجی/ عمودی و برآیند در پرش – فرود رو به لترال در گروه های درمانی (مداخله و کنترل) قبل و بعد از مداخله
- تعیین میانگین زمان رسیدن به ثبات در جهات قدامی- خلفی/ داخلی- خارجی و برآیند در پرش – فرود رو به لترال در گروه های درمانی (مداخله و کنترل) قبل و بعد از مداخله
- تعیین میانگین مسافت طی شده در تست پرش تک پا در گروه های درمانی (مداخله و کنترل) قبل و بعد از مداخله
- تعیین میانگین زمان سپری شده در تست پرش جانبی در گروه های درمانی (مداخله و کنترل) قبل و بعد از مداخله
- تعیین میانگین میزان ترس از حرکت در گروه های درمانی (مداخله و کنترل) قبل و بعد از مداخله

**اهداف تحلیلی**

- مقایسه میانگین شاخص ثبات در جهات قدامی- خلفی/ داخلی- خارجی/ عمودی و برآیند در پرش – فرود رو به لترال در گروه های درمانی (مداخله و کنترل) بین قبل و بعد از مداخله
- مقایسه میانگین شاخص ثبات در جهات قدامی- خلفی/ داخلی- خارجی/ عمودی و برآیند در پرش – فرود رو به لترال بین گروه های درمانی (مداخله و کنترل) بعد از مداخله
- مقایسه میانگین زمان رسیدن به ثبات در جهات قدامی- خلفی/ داخلی- خارجی و برآیند در پرش – فرود رو به لترال در گروه های درمانی (مداخله و کنترل) بین قبل و بعد از مداخله
- مقایسه میانگین زمان رسیدن به ثبات در جهات قدامی- خلفی/ داخلی- خارجی و برآیند در پرش – فرود رو به لترال بین گروه های درمانی (مداخله و کنترل) بعد از مداخله
- مقایسه میانگین مسافت طی شده در تست پرش تک پا در گروه های درمانی (مداخله و کنترل) بین قبل و بعد از مداخله
- مقایسه مسافت طی شده در تست پرش تک پا بین گروه های درمانی (مداخله و کنترل) بعد از مداخله
- مقایسه زمان سپری شده در تست پرش جانبی در گروه های درمانی (مداخله و کنترل) بین قبل و بعد از مداخله
- مقایسه زمان سپری شده در تست پرش جانبی بین گروه های درمانی (مداخله و کنترل) بعد از مداخله
- مقایسه میانگین میزان ترس از حرکت در گروه های درمانی (مداخله و کنترل) بین قبل و بعد از مداخله
- مقایسه میانگین میزان ترس از حرکت بین گروه های درمانی (مداخله و کنترل) بعد از مداخله

**6. اهداف کاربردی**

در صورتی که exergame بتواند نتایج بهتری بهمراه داشته باشد میتوان این روش را برای استفاده در کلینیک و محیط های ورزشی پیشنهاد داد زیرا این روش در مقایسه با تمرینات سنتی بسیار سرگرم کننده و لذت بخش هستند و امکان دنبال کردن آن برای افراد در شرایط مختلف و حتی بدون نظارت درمانگر امکان پذیر است. بعبارتی در صورتی که در این مطالعه exergame نتایج مطلوبی کسب کند میتواند به منظور بهبود بخشیدن کنترل پاسچرال داینامیک و بکارگیری استراتژیهای مناسب در حرکت پرش – فرود که یکی از کارهای رایج در فعالیت های ورزشی است و همچنین یک مکانیسم رایج آسیب مچ پا است مورد استفاده قرار گیرد. چنانچه گروه های درمانی تفاوت چندانی با هم نشان ندادند میتوان اینگونه برآورد کرد که هر دو درمان اثربخش و کارآمد هستند و از این رو با توجه به شرایط و امکانات میتوان از هر دو روش برای بهبود استراتژیهای کنترل پاسچرال بهره برد.

**7. نوع مطالعه**

این پژوهش از نوع کارآزمایی بالینی تصادفی یک سور کور به منظور بررسی اثرات خالص و متقابل عامل درون گروهی مرحله زمانی (قبل و بعد از مداخله) و بین گروهی نوع درمان (تمرینات تعادلی و Exergame)

**8. جمعیت مورد مطالعه**

جامعه هدف: ورزشکاران مبتلا به بی ثباتی مچ پا

جامعه مورد مطالعه: ورزشکاران مبتلا به بی ثباتی مچ پای در دسترس

**9. روش نمونه گیری و تعیین حجم نمونه**

نمونه ها بصورت نمونه گیری غیر احتمالی ساده، داوطلبانه و براساس ضوابط ورود و خروج انتخاب خواهند شد.

با توجه به مطالعه محمدی و متغیر زمان سپری شده در تست پرش جانبی برای اینکه اثربخشی exergame در قبل و بعد از درمان مشخص شود به 6 نفر در گروه نیاز هست ( 0.05=α و (Power= 0.95 و همچنین برای اینکه تفاوت بین گروه ها بعد از مداخله مشخص گردد به 13 نفر در هر گروه نیاز هست ( 0.05=α و(Power= 0.8 (31). از این رو 13 نفر در هر گروه تخمین زده میشود.

**10. شرایط ورود به مطالعه**

- بازه سنی 40-18 سال
- هر دو جنس
- ورزشکار (والیبالیست، بسکتبالیست و یا فوتبالیست) با داشتن فعالیت فیزیکی متوسط تا شدید (حداقل 3 بار در هفته و هر بار بیش از 30 دقیقه) (53)
- داشتن سابقه پیچ خوردگی مچ پا به صورت یکطرفه، حداقل 12 ماه قبل از ورود به مطالعه (از آخرین پیچ خوردگی بیش از 3 ماه در هنگام ورود به مطالعه گذشته باشد)
- تجربه اپیزود های مکرر خالی کردن مچ پا یا احساس بی ثباتی و داشتن علایمی نظیر ضعف، کاهش عملکرد در طول یک سال گذشته (54)
- داشتن امتیاز 24 یا پایین تر برای پرسشنامه بی ثباتی مچ پا کامبرلند.

**توضیحات:** این پرسشنامه از 9 سوال تشکیل شده است که جمع امتیازبندی سوالات آن 30 میباشد که امتیاز بیشتر نشان دهنده ی وجود ثبات بالا در مچ پا می باشد. نسخه فارسی پرسشنامه بی‌ثباتی مچ پای کامبرلند، می‌تواند به ‌عنوان ابزاری پایا برای تشخیص بی‌ثباتی و سنجش تغییرات ناشی از مداخلات درمانی در ورزشکاران مبتلا به بی‌ثباتی فانکشنال مچ پا مورد استفاده قرار گیرد (55).

- عدم سابقه عمل جراحی یا شکستگی در مچ پا و یا اندام تحتانی براساس گزارش خود بیمار
- سابقه تمرین با بازیهای ویدیویی را نداشته باشد.

**11. شرایط خروج از مطالعه**

- توانایی درک یا انجام مانور های مورد نیاز را نداشته باشند
- حین انجام تست ها درد داشته باشند
- آن دسته از شرکت کنندگان که به هر علتی تمایل به ادامه همکاری را نداشته باشند
- غیبت در دو جلسه متوالی و سه جلسه غیر متوالی از جلسات درمانی

**12. روش اجرای طرح**

پس از دریافت کد اخلاق و ثبت پروپوزال در سامانه کارآزمایی بالینی که از شروط اصلی اجرای پژوهش است، بنرهایی برای فراخوان افراد واجد شرایط در سه کلینیک فیزیوتراپی ورزشی قرار می دهیم از بین افرادی که داوطلبانه تقاضای شرکت در طرح را داشته اند، به شرکت کنندگانی که شرایط لازم برای شرکت در مطالعه را احراز نموده اند، توضيحاتی در مورد نحوه انجام تحقيق داده می شود تا فرد شرکت کننده آگاهی کاملی در مورد چگونگی انجام مراحل داشته باشد و درصورت تمايل، موافقت آگاهانه خود را به صورت امضای رضايتنامه کتبی که مورد تائید کمیته اخلاق دانشگاه علوم پزشکی تهران قرارگرفته است اعلام نمايد. در صورت غیبت در دو جلسه درمانی پشت سر هم و یا عدم تمایل شخصی افراد در هر مرحله از طرح افراد از گروه حذف می شوند. پیش از شروع طرح معیار های ورود به صورت یک پرسشنامه کتبی در اختیار افراد قرار گرفته و در صورت داشتن تمامی شرایط افراد وارد طرح پژوهشی می شوند. به صورت تصادفی افراد در دو گروه مداخله و کنترل قرار می گیرند. درمانها به مدت 12 جلسه (4 هفته و هر هفته 3 جلسه) انجام مي شود. بطور کلی در این مطالعه ارزیابی ها قبل و بعد از اتمام درمان انجام میگیرد.

- **ارزیابی ها**

ارزیابی ها قبل و بعد از پایان دوره درمانی انجام می شوند. ارزیابی ها شامل 1 تست آزمایشگاهی، 2 تست بالینی و تکمیل یک پرسشنامه میباشد.

- **تست آزمایشگاهی**

در این تست از دستگاه صفحه نیرو (Bertec Corporation, Columbus, OH, USA) با فرکانس نمونه برداری 500 هرتز استفاده میشود. به منظور اندازه گیری ثبات پاسچرال داینامیک از تست پرش- فرود رو به لترال استفاده میشود. از این تست، شاخص ثبات و زمان رسیدن به ثبات در جهات مختلف استخراج میشود.

ابتدا به منظور تعیین میزان حداکثر پرش افقی، از شرکت کننده خواسته میشود تا 3 بار پرش رو به لترال را با حداكثر مسافت ممكن انجام دهد. حداكثر مسافت طی شده به عنوان میزان حداکثر پرش افقی در نظر گرفته میشود. سپس به خاطر بي خطر بودن براي فرد، 75 درصد از آن براي انجام پرش روی صفحه نیرو در نظر گرفته مي شود. بعبارتی هر فرد در فاصله ی 75 درصد حداکثر پرش افقی نسبت به مرکز صفحه نیرو روی دو پا قرار میگیرد در حالي که سر رو به جلو و دستها روی مفاصل هیپ قرار گرفته اند سپس روی پایی که دچار بی ثباتی است، فرود می آید. از شرکت کننده خواسته میشود بعد از لحظه فرود سعی کند هر چه سریع تر ثبات خود را بازیابد (تصویر 1).

لازم به ذکر است قبل از انجام تست اصلي تعدادي پرش به صورت تمريني انجام مي گیرد. همچنین در صورتي كه شرکت کننده ضمن فرود از پرش نتواند تعادل خود را حفظ کند و يا پای مقابلش در پرش یا فرود مداخله داشته باشد و یا فرودش همراه با يك جهش كوچك اضافي باشد يا نوسان زيادي در دستها، تنه و پاي مقابلش اتفاق افتد كه سبب بلند شدن پاي مورد تست از روي صفحه نيرو شود، آن تست حذف و مجدد تكرار ميشود. داده هاي خام حاصل از 3 پرش موفق شامل تغییرات نيروي عكس‌العمل زمين در جهات (x, y, z)، به مدت 15 ثانيه پس از فرود روي صفحه نيرو ثبت میگردد. فاصله هر تکرار 1 دقیقه میباشد.


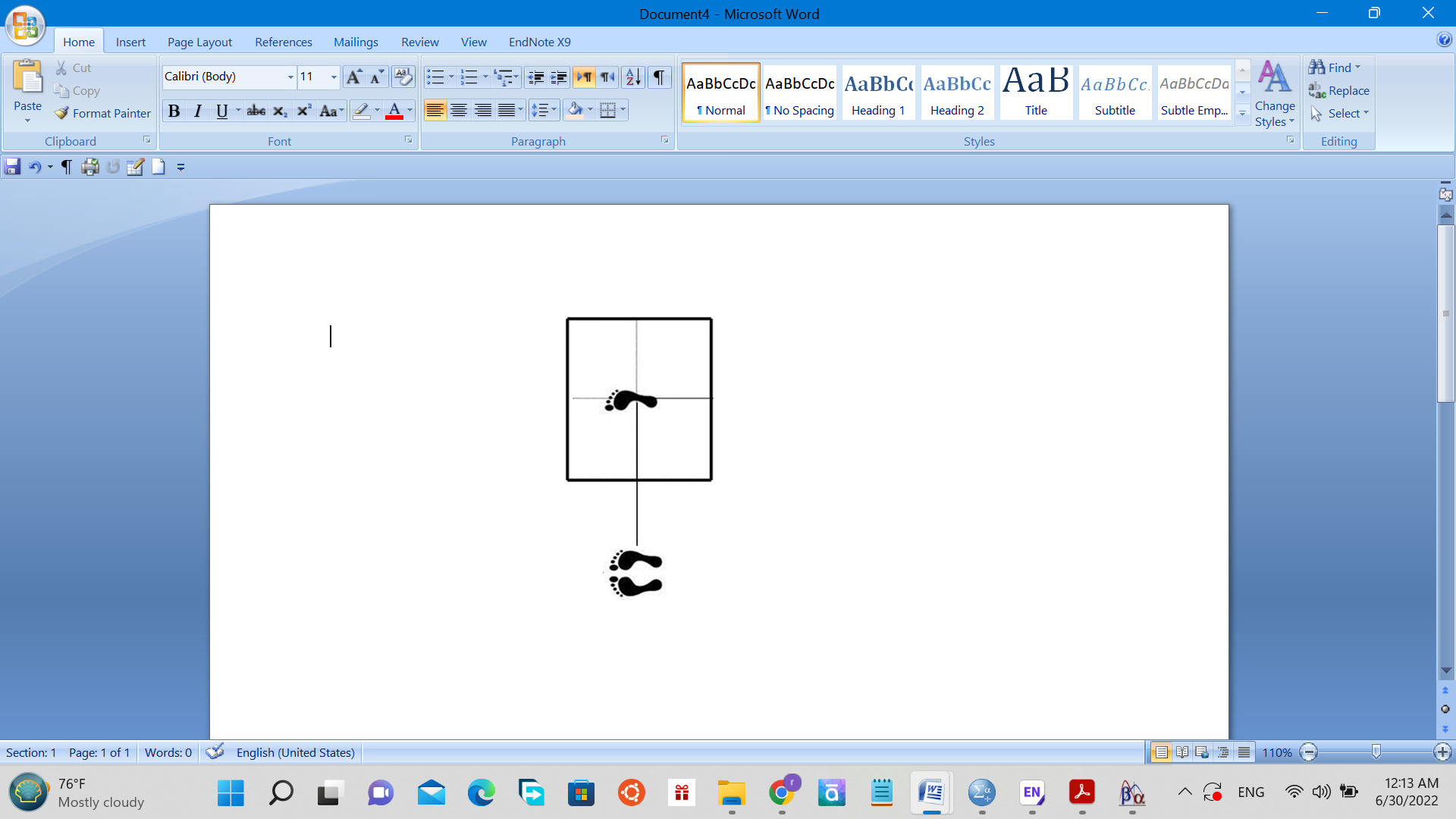


تصویر 1. پرش رو به لترال

- **شاخص ثبات:**

شاخص ثبات در جهت داخلي-خارجي و قدامي-خلفي، بر طبق فرمول زیر به ترتیب با اندازه گیری ميزان انحراف مولفه X و Y نيروي عكس‌العمل زمين از نقطه صفر بدست می آیند (فرمول 1و 2). همچنین شاخص ثبات در جهت عمودي، با اندازه گیری ميزان انحراف مولفه Z نيروي عكس العمل زمين از وزن فرد بدست می آید (فرمول 3). شاخص ثبات پاسچرال داینامیک، تركيبي از شاخص هاي ثبات پاسچرال در جهت داخلي - خارجي، قدامي - خلفي و عمودي است كه به تغييرات در هر سه جهت حساس است (فرمول 4). همانطور که در فرمولها مشاهده میشود مقادیر براساس وزن هر فرد نرمالایز میشود تا مقایسه بین افراد امکانپذیر شود. سپس میانگین 3 تکرار به عنوان داده نهایی لحاظ میگردد (56). لازم به ذکر است از لحظه فرود (لحظه ای که مولفه عمودی نيروي عكس العمل زمين از 5 درصد وزن بدن فراتر رود) تا 3 ثانیه بعد از آن برای محاسبات استفاده میشود زیرا این زمان تا حد امکان بهترین انتخاب برای تقلید از عملکرد ورزشی می باشد (36, 56).


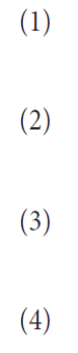


- **زمان رسیدن به ثبات:**

برای محاسبه زمان رسیدن به ثبات، ابتدا یک سری زمانی^[[14]](#footnote-15)^ با میانگین‌گیری متوالیِ^[[15]](#footnote-16)^ نیروهای عکس العمل نرمالایز شده در جهات داخلی- خارجی، قدامی- خلفی و عمودی بدست میآید. در این روش بر طبق فرمول زیر، بصورت متوالی 1 داده از نیروی عکس العمل به داده های قبلی اضافه شده و میانگین گیری انجام میشود (فرمول 5). در نتیجه یک سری زمانی از میانگین‌گیری متوالی نیروهای عکس العمل بدست میآید. علاوه براین، انحراف معیار و میانگین سری زمانی نیروهای عکس العمل نرمالایز شده در جهات داخلی- خارجی، قدامی- خلفی در 3 ثانیه اول بعد از فرود محاسبه و "انحراف معیار 25/0 ± میانگین" بعنوان آستانه درنظر گرفته میشود. زمانی که سری زمانی از میانگین‌گیری متوالی نیروهای عکس العمل در محدوده بین حد بالا و پایین آستانه قرار گیرد به معنای ثبات میباشد. از این رو لحظه ای که سری زمانی با میانگین‌گیری متوالی در این محدوده قرار گیرد لحظه ثبات درنظر گرفته شده و فاصله بین این لحظه و لحظه فرود به عنوان زمان رسیدن به ثبات تعریف میشود. برای نیروهای عکس العمل در جهت عمودی، آستانه براساس" 5% ± وزن فرد" تعریف میشود (تصاویر 2 و 3). زمان رسیدن به ثبات برآیند نیز از طریق فرمول 6 محاسبه میشود. سپس میانگین 3 تکرار به عنوان داده نهایی برای هر جهت لحاظ میگردد. لازم به ذکر است برای تعیین وزن بدن، از شرکت کننده خواسته میشود به مدت 5 ثانیه روی صفحه نیرو روی یک پا بایستد و میانگین نیروی عکس العمل عمودی در طی این 5 ثانیه ایستادن به عنوان وزن فرد تلقی میشود (36).


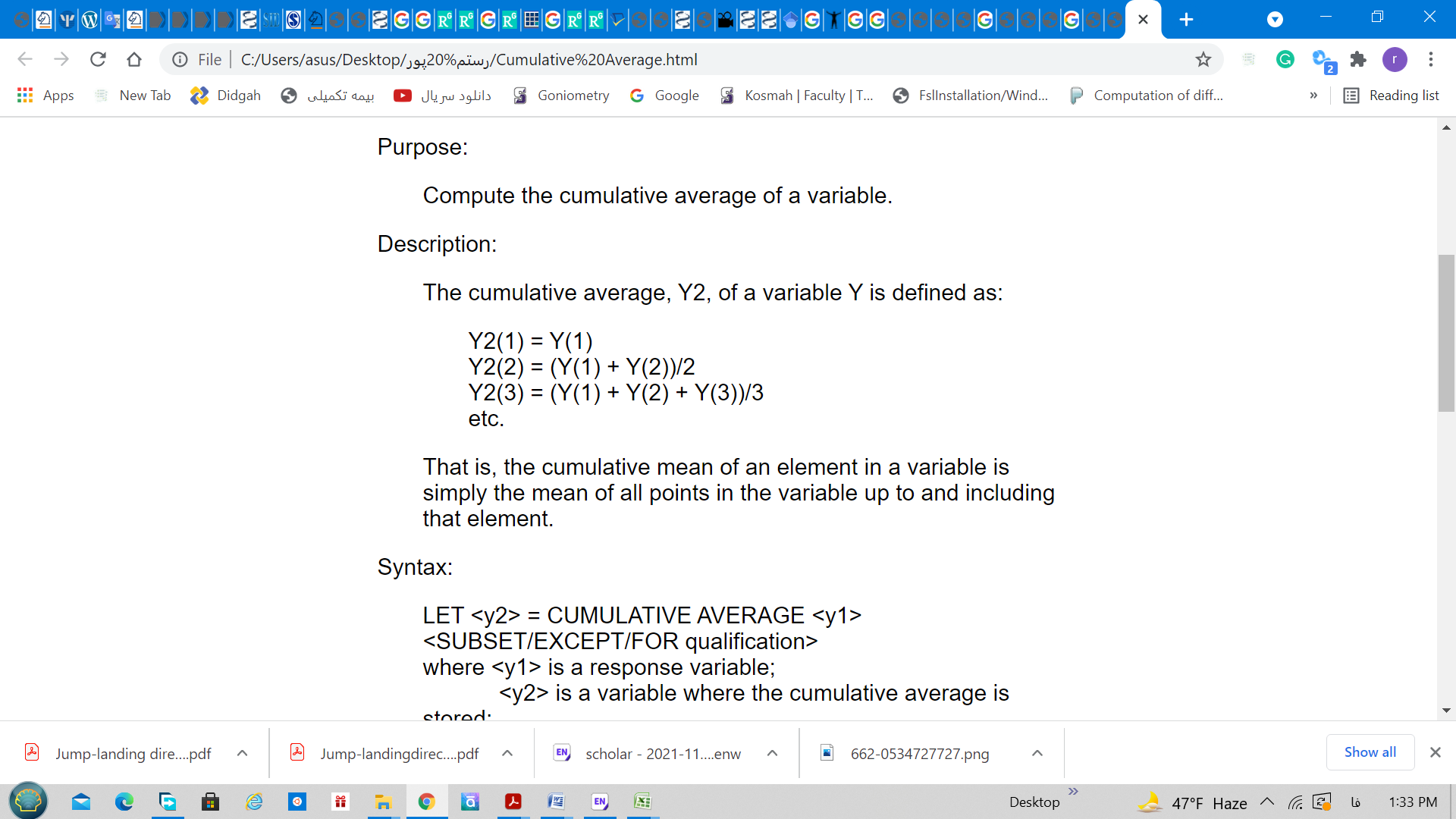


(5)

(6)


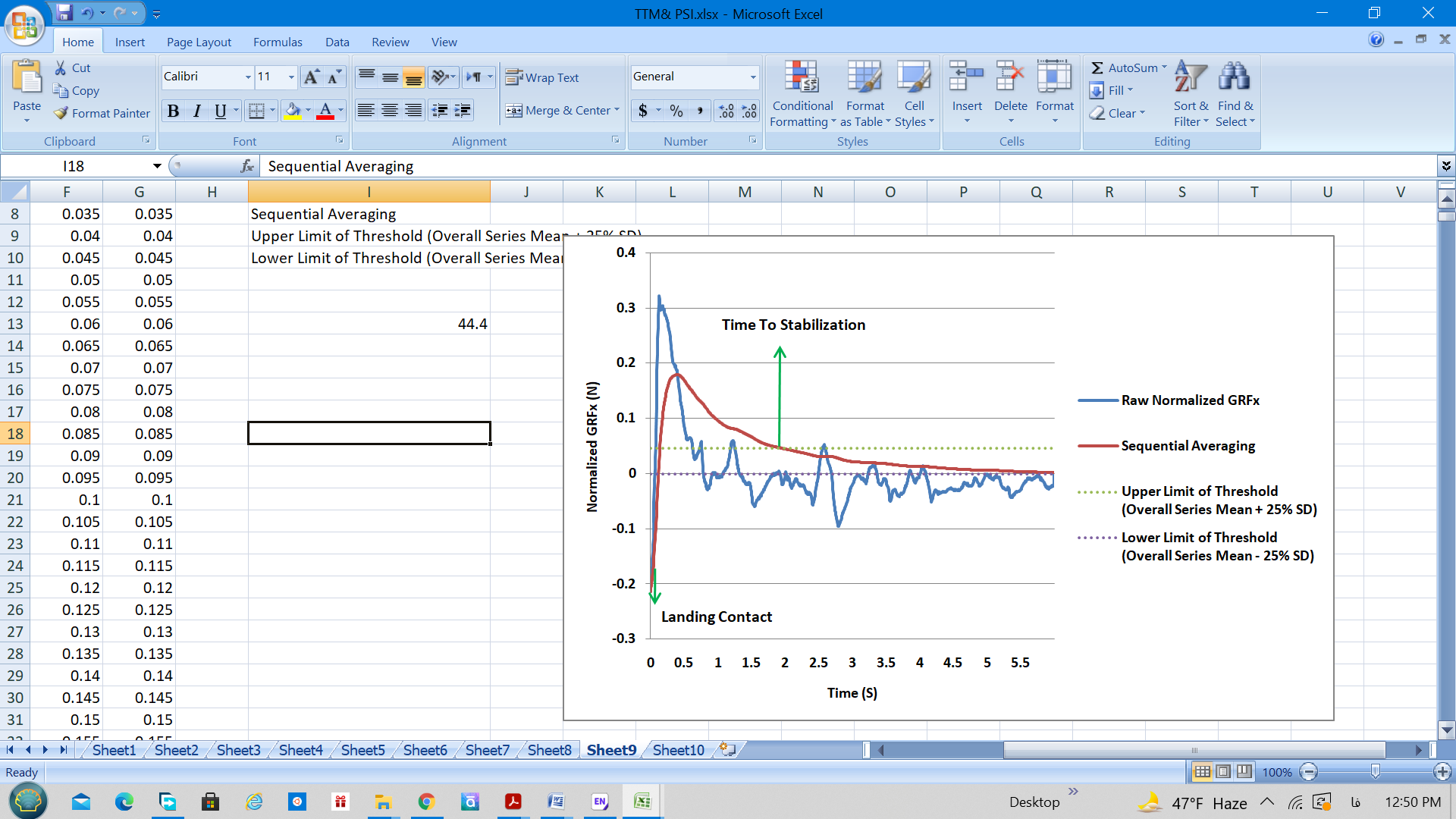


تصویر 2. زمان رسیدن به ثبات در جهت داخلی - خارجی


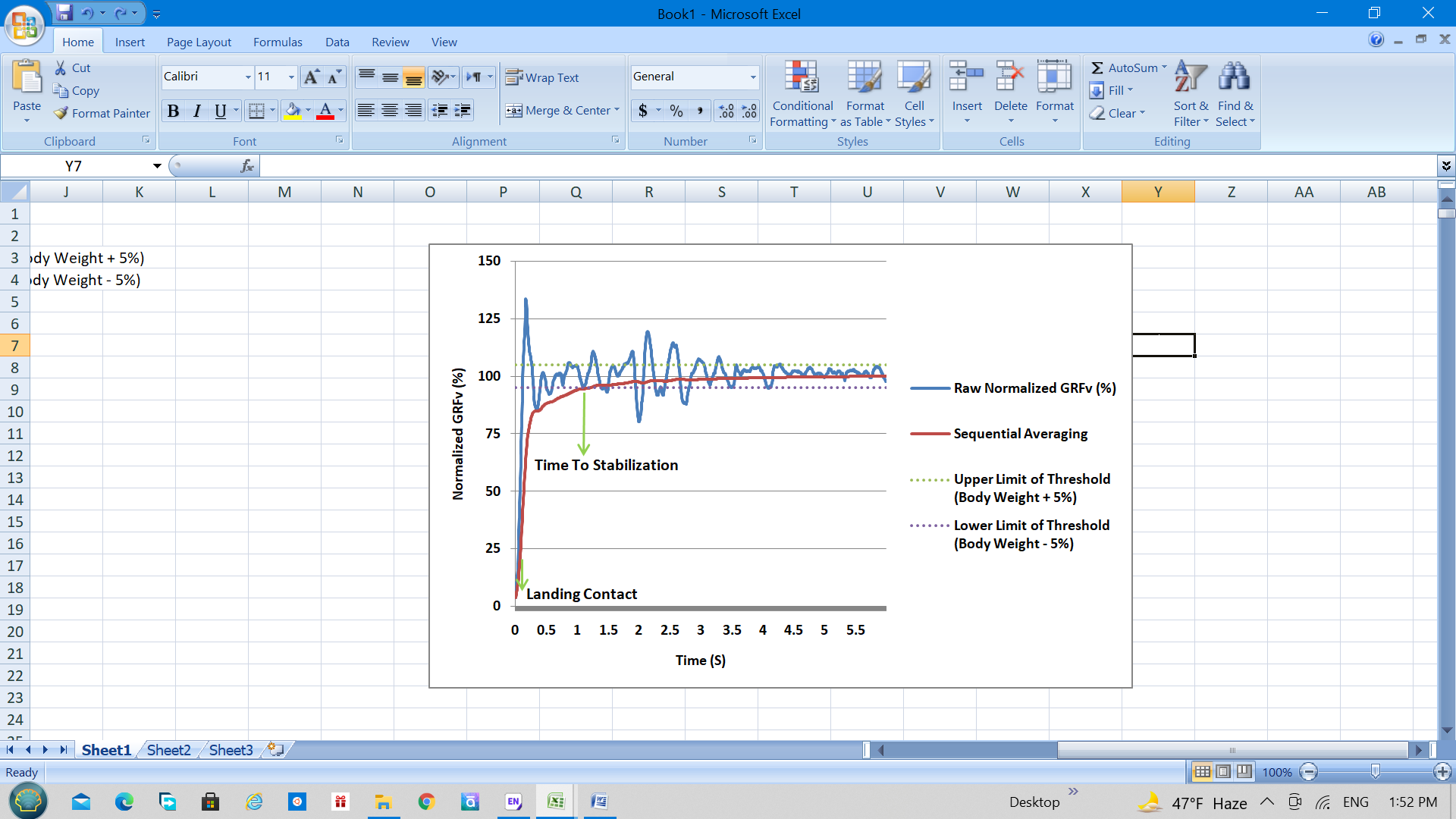


تصویر 3. زمان رسیدن به ثبات در جهت عمودی

- **تست بالینی (عملکرد فیزیکی)**

در این مطالعه از تست پرش تک پا^[[16]](#footnote-17)^ و پرش جانبی^[[17]](#footnote-18)^ استفاده میشود. در تست پرش تک پا از شرکت کننده خواسته میشود تا با حداکثر فاصله ای که میتواند رو به جلو پرش کند. به وسیله مترنواری فاصله از موقعیت انگشتان پا روی خط شروع تا انتهای پرش اندازه گیری میشود. این تست دارای ICC=0.96 و SEM=4.56 میباشد (51). در تست پرش جانبی از شرکت کننده خواسته میشود به صورت طرفی روی پای آسیب دیده در فاصله 30 سانتیمتری فرود آید. 10 بار این روند تکرار میشود. لازم به ذکر است یک تکرار شامل رفت و برگشت به نقطه شروع میباشد. از شرکت کنندگان خواسته میشود تا حد امکان با سرعت این کار را انجام دهند و زمان سپری شده توسط کرونومتر اندازه گیری میشود. این تست دارای ICC=0.84 و SEM=2.10 میباشد (51).

- **ترس از حرکت**

در این مطالعه برای بررسی ترس از حرکت از پرسشنامه تمپا استفاده می شود. این پرسشنامه شامل یازده آیتم است برای هر سوال 4 گزینه در نظر گرفته شده که نمره هر گزینه به صورت کاملا مخالفم=1، مخالفم =2، موافقم =3 و کاملا موافقم = 4 است. در پایان فرد می تواند نمراتی بین 11 تا 44 در این پرسشنامه کسب کند و میزان ترس از حرکت سنجیده می شود. این تست دارای ICC=0.81 و SEM =2.54 می باشد (52, 57). در قسمت "روش و ابزار گردآوری داده ها" جزییات مربوط به پرسشنامه آورده شده است.

- **درمان ها**

پس از اتمام ارزیابی اولیه، افراد به طور تصادفی به دو گروه تقسیم می شوند که با استفاده از توالی randomization ازطریق سایت randomization.com و به روش balanced block randomization انجام خواهد شد. سایز بلوک ها 4 تایی می باشد. همچنین به منظور allocation concealment از پاکت های مهر موم شده و شماره گذاری شده استفاده می شود که توسط منشی در اختیار افراد شرکت کننده قرار میگیرد. دوره درمان در هر دو گروه 12 جلسه و بصورت 3 بار در هفته است. ارزیابی ها قبل و بعد از اتمام درمان انجام میگیرند.

افراد شرکت کننده Blind نیستند و با توجه به طراحی مطالعه امکان Blind بودن تراپیست ها وجود ندارد و فرد آنالیزگر Blind می شود به این ترتیب که نفر سومی که خارج از مطالعه میباشد داده های ثبت شده را کد گذاری کرده به گونه ای که مفهوم کدها مشخص نمیباشد. سپس داده ها را در اختیار آنالیزگر قرار داده و آنالیزگر داده ها را باکمک نرم افزار پردازش میکند. سپس همان نفر سوم که کدگذاری کرده بود داده ها را وارد SPSS میکند.

- **گروه کنترل (تمرینات روتین)**

دوره درمان 12 جلسه (3 بار در هفته) و هر جلسه 1 ساعت است جدول زیر سیر پیشرفت درمان را نشان میدهد (10, 58-60).

| **نوع تمرینات** | **روش اجرا و شدت تمرینات** | **پیشرفت** |
| --- | --- | --- |
| ایستادن روی یک پا و پرتاب توپ | ایستادن روی یک پا در دو حالت چشم باز و چشم بسته انجام میشود.  3 تکرار | ایستادن روی یک پا با چشمان باز و بسته هر کدام دارای 7 سطح دشواری میباشد و در صورتی فرد از سطحی به سطح دیگر پیشرفت پیدا میکند که بتواند در هر سطح 3 تکرار بدون خطا انجام دهد.   - **سطوح دشواری ایستادن با چشمان باز:**   1) ایستادن روی سطح سفت برای مدت 60 ثانیه با دستها روی قفسه سینه  2) ایستادن روی سطح فوم برای مدت 30 ثانیه با دستها روی قفسه سینه  3) ایستادن روی سطح فوم برای مدت 60 ثانیه با دستها روی قفسه سینه  4) ایستادن روی سطح فوم برای مدت 90 ثانیه با دستها روی قفسه سینه  5) ایستادن روی سطح فوم برای مدت 30 ثانیه با دستها روی قفسه سینه و 20 پرتاپ توپ با وزن 6 پوند  6) ایستادن روی سطح فوم برای مدت 60 ثانیه با دستها روی قفسه سینه و 20 پرتاپ توپ با وزن 6 پوند  7) ایستادن روی سطح فوم برای مدت 90 ثانیه با دستها روی قفسه سینه و 20 پرتاپ توپ با وزن 6 پوند   - **سطوح دشواری ایستادن با چشمان بسته:**   1) ایستادن روی سطح سفت برای مدت 30 ثانیه با دستهای باز  2) ایستادن روی سطح سفت برای مدت 30 ثانیه با دستها روی قفسه سینه  3) ایستادن روی سطح سفت برای مدت 60 ثانیه با دستها روی قفسه سینه  4) ایستادن روی سطح فوم برای مدت 30 ثانیه با دستهای باز  5) ایستادن روی سطح فوم برای مدت 30 ثانیه با دستها روی قفسه سینه  6) ایستادن روی سطح فوم برای مدت 60 ثانیه با دستها روی قفسه سینه  7) ایستادن روی سطح فوم برای مدت 90 ثانیه با دستها روی قفسه سینه   - **خطاها**:   1) خوردن پای مقابل به زمین  2) حرکات اضافی تنه (30 درجه فلکشن طرفی)  3) بلند شدن دستها از روی قفسه سینه هنگام ایستادن  4) تکیه دادن پای مقابل کنار پای ایستا |
| شوت کردن توپ پرتاب شده توسط آزمونگر | ایستادن روی پا در حالت چشم باز و شوت کردن توپ  3 تکرار | ایستادن روی پا با چشمان باز که دارای 5 سطح دشواری میباشد و در صورتی فرد از سطحی به سطح دیگر پیشرفت پیدا میکند که بتواند در هر سطح 3 تکرار بدون خطا انجام داد.   - **سطوح دشواری:**   1) ایستادن روی دو پا روی سطح سفت  2) ایستادن روی یک پا روی سطح سفت  3) ایستادن روی دو پا روی سطح بی ثبات (مینی ترامپولین)  4) ایستادن روی یک پا روی سطح بی ثبات (مینی ترامپولین)  5) مشابه حالت قبل با این تفاوت که فرد 45 درجه نسبت به آزمونگر مایل می ایستد (بین صفحه فرونتال و ساجیتال)   - **خطاها**:   1) خوردن پای مقابل به زمین  2) حرکات اضافی تنه (30 درجه فلکشن طرفی)  3) تکیه دادن پای مقابل کنار پای ایستا |
| hop to stabilization روی یک پا | همانطور که شکل نشان میدهد 3 هدف با فاصله های 18، 27 و36 اینچ در جهات قدامی/خلفی، داخلی/خارجی، قدامی خارجی/ خلفی داخلی و قدامی داخلی/خلفی خارجی نسبت به پای راست روی زمین رسم میشود. از افراد خواسته میشود که از نقطه شروع به سمت هر کدام از اهداف پریده و سعی کند ثبات خود را حفظ کند. سپس به نقطه آغازین پریده و ثبات خود را حفظ میکند.  10 تکرار در هر جهت | هر مسیر دارای 7 سطح دشواری میباشد و در صورتی فرد از سطحی به سطح دیگر پیشرفت پیدا میکند که بتواند در هر سطح 10 تکرار بدون خطا انجام داد.   - **سطوح دشواری:**   1) هدف با فاصله 18 اینج قرار داشته و به فرد اجازه داده میشود که برای حفظ ثبات در هنگام فرود آمدن از بازوها استفاده کند.  2) هدف با فاصله 18 اینج قرار داشته و هنگام فرود آمدن، دستها بر روی لگن قرار دارد.  3) هدف با فاصله 27 اینج قرار داشته و به فرد اجازه داده میشود که برای حفظ ثبات در هنگام فرود آمدن از بازوها استفاده کند.  4) هدف با فاصله 27 اینج قرار داشته و هنگام فرود آمدن، دستها بر روی لگن قرار دارد.  5) هدف با فاصله 36 اینج قرار داشته و به فرد اجازه داده میشود که برای حفظ ثبات در هنگام فرود آمدن از بازوها استفاده کند.  6) هدف با فاصله 36 اینج قرار داشته و هنگام فرود آمدن، دستها بر روی لگن قرار دارد.  7) پرش به سمت هدف با فاصله 36 اینج از روی یک پله 6 اینچی انجام میگیرد.   - **خطاها:**   1) خوردن پای مقابل به زمین  2) حرکات اضافی تنه (30 درجه فلکشن طرفی)  3) بلند شدن دستها از روی لگن هنگام فرود آمدن  4) تکیه دادن پای مقابل کنار پای ایستا  5) عدم فرود آمدن مناسب روی هدف |
| Hop to stabilization and reach | مشابه قبلی با این تفاوت که پس از پرش و حفظ ثبات، فرد دستها را به سمت نقطه آغازین دراز میکند و هنگام برگشت نیز دستها را به سمت نقطه هدف دراز میکند.  5 تکرار در هر جهت | پیشرفت مانند قبلی است با این تفاوت که برای پیشرفت از هر سطح به سطح دیگر فرد بایستی بتواند در هر سطح 5 تکرار بدون خطا انجام دهد.  خطاها مانند قبل است به علاوه اگر فرد از دراز کردن پا به همراه دستها برای حفظ ثبات استفاده کند خطا محسوب میشود. |

- **گروه مداخله (exergame)**

در این گروه شرکت کننده بر روی صفحه تعادلی wii (Nintendo Co.Ltd., Kyoto, Japan) می ایستد و تعادل خود را با انتقال وزن حین تمرینات مختلف حفظ میکند. در این مطالعه، بازیهای Single Leg Extension, Torso Twist, Single Leg Twist, Sideways Leg Lift, Rowing Squat, Table Tilt, Penguin Fishing, Soccer Heading, Tightrope Walk, and Snowboard Slalom مورد استفاده قرار میگیرد. در هر جلسه هر بازی تقریبا 6 دقیقه انجام میشود که مجموعا 1 ساعت میشود. دوره درمان 12 جلسه (3 جلسه در هفته) میباشد. به طور کلی هر بازی دارای 3 سطح دشواری (مبتدی، پیشرفته و متخصص) است که هر سطح خود دارای 4 زیرسطح (بی ثبات، آماتور، حرفه ای و قهرمان) است. سطح فرد براساس تعداد ستاره ها مشخص میشود 1 ستاره (بی ثبات)، 2 ستاره (آماتور)، 3 ستاره (حرفه ای) و 4 ستاره (قهرمانی). در صورتی که شرکت کننده توانایی انجام بازی مربوط به یک سطح را داشته باشد به سطح بالاتر ارتقا پیدا می کند. همچنین ترتیب انتخاب بازیها در هر جلسه براساس میزان علاقه مندی شرکت کنندگان میباشد. توضیحات مربوط به بازیهای مورد استفاده در تصاویر 7 تا 12 قابل مشاهده است (23, 61).

- بازی Table tilt:

در این بازی، فرد با شیفت وزن روی تخته تعادل توپ را روی تخته شناور نگه داشته و سپس آن را داخل حفره می اندازد.

- بازی Penguin Fishing:

در این بازی، فرد با شیفت وزن به طرفین پنگوئن روی تکه یخ را حرکت داده و سعی می کرد تا حد ممکن ماهی صید کند.

- بازی Soccer Heading:

فرد با شیفت وزن به چپ و راست سعی می کرد کاراکتر بازی با سر به توپ ضربه بزند و بایستی مراقب می بود سر به سایر اجسام برخورد نکند.

- بازی Tightrope walk:

فرد با شیفت وزن در جهات مختلف و حفظ تعادل و همچنین گام برداشتن روی تخته تعادل کاراکتر بازی را روی طناب پیش می برد.

- بازی Single Leg Extension:

در این بازی فرد روی یک پا ایستاده و دستها و پاها را بصورت متقاطع حرکت میدهد و در عین حال سعی میکند تعادل خود را حفظ کرده و مرکز ثقل را روی دایره زرد رنگ نگه دارد.

- بازی Torso Twist:

فرد تنه را میچرخاند درحالیکه دستها را به اطراف حرکت میدهد و در عین حال سعی میکند تعادل خود را حفظ کرده و مرکز ثقل را در دایره زرد رنگ نگه دارد.

**14-5. روش و ابزار گردآوری داده ها**

- اطلاعات فردی به روش مصاحبه و ثبت در پرسشنامه
- وزن نمونه ها توسط ترازو
- قد نمونه ها توسط متر نواری
- بررسی عملکرد توسط تست های پرش تک پا و پرش جانبی و سنجش بوسیله مترنواری و کرونومتر
- بررسی شاخص ثبات و زمان رسیدن به ثبات در حرکت پرش- فرود با استفاده از دستگاه صفحه نیرو (Bertec Corporation, Columbus, OH, USA)
- بررسی ترس از حرکت توسط پرسشنامه تمپا انجام میگیرد. نسخه ی فارسی این پرسشنامه به صورت زیر است:

| ردیف | عبارات | كاملا مخالفم | مخالفم | موافقم | كاملا موافقم |
| --- | --- | --- | --- | --- | --- |
| **1** | وضعیت جسمی ام نشانگر آن است كه دارای مشكلی جدی هستم. |  |  |  |  |
| **2** | از این كه ممكن است طی انجام فعالیتی، به طور اتفاقی به خودم آسیب برسانم، می ترسم. |  |  |  |  |
| **3** | می ترسم در صورت انجام فعالیتی به خودم صدمه بزنم. |  |  |  |  |
| **4** | حادثه ای كه برایم پیش آمد، برای بقیه عمر، بدنم را در قبال خطرات آسیب پذیر كرده است. |  |  |  |  |
| **5** | درد هموار به این معنی است كه به جسمم آسیب رسانده ام. |  |  |  |  |
| **6** | اگر واقعا مشكل جدی در بدنم وجود نداشت، نمی بایست این همه درد می داشتم. |  |  |  |  |
| **7** | برای فردی با شرایط من انجام فعالیت های جسمی كار درستی نیست. |  |  |  |  |
| **8** | هیچكس نباید زمانی كه درد دارد، فعالیت كند. |  |  |  |  |
| **9** | چون ممكن است به راحتی آسیب ببینم، نمی توانم كارهایی را كه مردم عادی انجام می دهند، انجام دهم. |  |  |  |  |
| **10** | درد به من می گوید كه چه وقت باید فعالیتم را متوقف كنم، تا به خودم آسیب نرسانم. |  |  |  |  |
| **11** | دقت در انجام ندادن حركات غیر ضروری مطمئن ترین كاری است كه می توانم برای بهتر شدن دردم انجام دهم. |  |  |  |  |

هدف آن ارزیابی میزان ترس از حركت از دو بعد مختلف باور به آسیب دیدگی (سوالات 1-6) و اجتناب از فعالیت ( سوالات 7-11) می باشد. برای هر سوال 4 گزینه در نظر گرفته شده که نمره هر گزینه به صورت کاملا مخالفم=1، مخالفم =2، موافقم =3 و کاملا موافقم = 4 است . در پایان مجموع نمرات با جدول زیر تطابق داده می شود:

| **حد پایین نمره** | **حد متوسط نمرات** | **حد بالای نمرات** |
| --- | --- | --- |
| 11 | 27 | 44 |

حداقل امتیاز ممکن 11 و حداکثر 44 خواهد بود.

- نمره بین 11 تا 18 : ترس از حركت، كم است.
- نمره بین 18 تا 27 : ترس از حركت، متوسط است.
- نمره بالاتر از 27 : ترس از حركت، زیاد است

**15-5. روش تجزيه و تحليل داده ها**

ابتدا توزیع نرمال داده ها توسط تست shapiro wilkانجام میشود. در صورت توزیع نرمال داده ها از 2 way mixed ANOVA استفاده میشود. متغیرهای مستقل، گروه با دو سطح (مداخله و کنترل) و زمان با دو سطح (قبل و بعد) میباشد. در صورت عدم توزیع نرمال از معادل غیرپارامتریک استفاده میشود. برای بررسی اثر گروه از Mann-Whitney U test و برای بررسی زمان از Wilcoxon signed-rank test استفاده میشود.

**16-5. ملاحظات اخلاقی**

1. پس از کسب تاییدیه ازکمیته اخلاق دانشگاه علوم پزشکی تهران، تمام مراحل ارزیابی و انجام آزمون در دانشکده توانبخشی این دانشگاه انجام می گیرد.

2. قبل از اجرای آزمون، کلیه مراحل آن، روش و هدف انجام آن برای افراد شرح داده می شود و شرکت کنندگان فرم موافقت آگاهانه را مطالعه و تکمیل می کردند.

3. ابزارهای مورد استفاده دراین پژوهش به صورت غیرتهاجمی بوده و هیچ آسیب به افراد شرکت کننده وارد نمیشود.

4. به افراد شرکت کننده در این مطالعه اطمینان لازم داده میشود که ازآنها هیچ هزینه ای دریافت نخواهد شد و همه آزمون ها به صورت کاملا رایگان اجرا میشوند؛ و درصورت هرگونه هزینه احتمالی ناشی از شرکت دراین مطالعه، محقق متعهد به جبران آن خواهد بود.

5. آزمون ها به گونه ای هستند که باعث هیچ گونه افزایش شدت اختلال یا علایم بالینی در افراد شرکت کننده نخواهند شد و در صورت افزایش احتمالی اختلال، آزمون ها متوقف میشوند.

6. افراد شرکت کننده دراین مطالعه، اجازه دارند در هرمرحله‌ ای از تحقیق به هر علتی یا بدون علت مشخصی، از ادامه همکاری با این مطالعه، انصراف دهند.

7. به شرکت کنندگان اطمینان داده میشود که از اطلاعات جمع آوری شده از آن ها محافظت گردیده، اصل رازداری رعایت شده و از اطلاعات فقط جهت تجزیه و تحلیل آماری استفاده میشود.

1. Kosik KB, McCann RS, Terada M, Gribble PAJBJoSM. Therapeutic interventions for improving self-reported function in patients with chronic ankle instability: a systematic review. 2017;51(2):105-12.

2. Wright I, Neptune R, van den Bogert AJ, Nigg BJJob. The influence of foot positioning on ankle sprains. 2000;33(5):513-9.

3. Tanen L, Docherty CL, Van Der Pol B, Simon J, Schrader JJF, specialist a. Prevalence of chronic ankle instability in high school and division I athletes. 2014;7(1):37-44.

4. Hiller CE, Nightingale EJ, Lin C-WC, Coughlan GF, Caulfield B, Delahunt EJBjosm. Characteristics of people with recurrent ankle sprains: a systematic review with meta-analysis. 2011;45(8):660-72.

5. Brown CN, Padua DA, Marshall SW, Guskiewicz KMJJoat. Hip kinematics during a stop-jump task in patients with chronic ankle instability. National Athletic Trainers' Association, Inc; 2011. p. 461-7.

6. Hertel JJJoat. Functional anatomy, pathomechanics, and pathophysiology of lateral ankle instability. 2002;37(4):364.

7. Attenborough AS, Hiller CE, Smith RM, Stuelcken M, Greene A, Sinclair PJJSm. Chronic ankle instability in sporting populations. 2014;44(11):1545-56.

8. McKeon PO, Hertel JJJoat. Systematic review of postural control and lateral ankle instability, part I: can deficits be detected with instrumented testing? 2008;43(3):293-304.

9. Williams VJ, Nagai T, Sell TC, Abt JP, Rowe RS, McGrail MA, et al. Prediction of dynamic postural stability during single-leg jump landings by ankle and knee flexibility and strength. 2016;25(3):266-72.

10. Anguish B, Sandrey MAJJoAT. Two 4-week balance-training programs for chronic ankle instability. 2018;53(7):662-71.

11. Docherty CL, McLeod TCV, Shultz SJJCjosm. Postural control deficits in participants with functional ankle instability as measured by the balance error scoring system. 2006;16(3):203-8.

12. McKeon PO, Hertel JJBmd. Spatiotemporal postural control deficits are present in those with chronic ankle instability. 2008;9(1):1-6.

13. Benzing V, Schmidt MJJocm. Exergaming for children and adolescents: strengths, weaknesses, opportunities and threats. 2018;7(11):422.

14. Elena P, Demetris S, Christina M, Marios PJFiN. Differences Between Exergaming Rehabilitation and Conventional Physiotherapy on Quality of Life in Parkinson's Disease: A Systematic Review and Meta-Analysis. 2021;12:683385.

15. Adcock M, Thalmann M, Schättin A, Gennaro F, De Bruin EDJFian. A pilot study of an in-home multicomponent exergame training for older adults: feasibility, usability and pre-post evaluation. 2019;11:304.

16. Jorgensen MG, Laessoe U, Hendriksen C, Nielsen OBF, Aagaard PJJoGSABS, Sciences M. Efficacy of Nintendo Wii training on mechanical leg muscle function and postural balance in community-dwelling older adults: a randomized controlled trial. 2013;68(7):845-52.

17. Lin H, Han K, Ruan BJJoHE. Effect of Virtual Reality on Functional Ankle Instability Rehabilitation: A Systematic Review. 2021;2021.

18. de Bruin ED, Schoene D, Pichierri G, Smith STJZfGuG. Use of virtual reality technique for the training of motor control in the elderly. 2010;43(4):229-34.

19. Holden MK. Virtual environments for motor rehabilitation: review. CyberPsychol Behav 2005; 8 (3): 187–211.

20. Schultheis MT, Rizzo AAJRp. The application of virtual reality technology in rehabilitation. 2001;46(3):296.

21. Stott I, Sanders DJIJoRR. The use of virtual reality to train powered wheelchair users and test new wheelchair systems. 2000;23(4):321-6.

22. dos Santos Mendes FA, Pompeu JE, Lobo AM, da Silva KG, de Paula Oliveira T, Zomignani AP, et al. Motor learning, retention and transfer after virtual-reality-based training in Parkinson's disease–effect of motor and cognitive demands of games: a longitudinal, controlled clinical study. 2012;98(3):217-23.

23. Khanmohammadi R, Olyaei G, Talebian S, Hadian MR, Hossein B, Aliabadi SJD, et al. The effect of video game-based training on postural control during gait initiation in community-dwelling older adults: a randomized controlled trial. 2021:1-8.

24. Valentina M, Ana Š, Valentina M, Martina Š, Željka K, Mateja ZJACC. Virtual reality in rehabilitation and therapy. 2013;52(4.):453-7.

25. Gioftsidou A, Vernadakis N, Malliou P, Batzios S, Sofokleous P, Antoniou P, et al. Typical balance exercises or exergames for balance improvement? 2013;26(3):299-305.

26. Kim A, Darakjian N, Finley JM. Walking in fully immersive virtual environments: an evaluation of potential adverse effects in older adults and individuals with Parkinson's disease. J Neuroeng Rehabil. 2017;14(1):16.

27. Satyen L, Ohtsuka K. Strategies to Develop Dual Attention Skills Through Video Game Training: Ashgate; 2001.

28. Kim K-J, Heo MJJoB, Rehabilitation M. Comparison of virtual reality exercise versus conventional exercise on balance in patients with functional ankle instability: a randomized controlled trial. 2019;32(6):905-11.

29. Kim K-J, Jun H-J, Heo MJJopts. Effects of Nintendo Wii Fit Plus training on ankle strength with functional ankle instability. 2015;27(11):3381-5.

30. Punt I, Ziltener JL, Monnin D, Allet LJSjom, sports si. W ii F it™ exercise therapy for the rehabilitation of ankle sprains: Its effect compared with physical therapy or no functional exercises at all. 2016;26(7):816-23.

31. Mohammadi N, Hadian M-R, Olyaei G-RJCR. Comparison of the effects of Wii and conventional training on functional abilities and neurocognitive function in basketball-players with functional ankle instability: Matched randomized clinical trial. 2021;35(10):1454-64.

32. Mohammadi N, Hadian M-R, Olyaei G-RJJoBP, Engineering. The Effect of Wii Training on Neurocognitive Function in Athletes with Functional Ankle Instability: Matched Randomized Clinical Trial. 2021.

33. Shousha TM, Abo-zaid NA, Hamada HA, Abdelsamee MYA, Behiry MAJDoBFoPT. Virtual reality versus Biodex training in adolescents with chronic ankle instability: a randomized controlled trial. 2021.

34. Head PL, Kasser R, Appling S, Cappaert T, Singhal K, Zucker-Levin AJPTiS. Anterior cruciate ligament reconstruction and dynamic stability at time of release for return to sport. 2019;38:80-6.

35. Webster KA, Gribble PAJJoat. Time to stabilization of anterior cruciate ligament–reconstructed versus healthy knees in National Collegiate Athletic Association Division I female athletes. 2010;45(6):580-5.

36. Wikstrom EA, Tillman MD, Smith AN, Borsa PAJJoat. A new force-plate technology measure of dynamic postural stability: the dynamic postural stability index. 2005;40(4):305.

37. Krupenevich RL, Pruziner AL, Miller RHJM, sports si, exercise. Knee Joint Loading during Single-Leg Forward Hopping. 2017;49(2):327-32.

38. Shiravi Z, Shadmehr A, Moghadam ST, Moghadam BAJM, ligaments, journal t. Comparison of dynamic postural stability scores between athletes with and without chronic ankle instability during lateral jump landing. 2017;7(1):119.

39. Wikstrom EA, Tillman MD, Borsa PAJM, sports si, exercise. Detection of dynamic stability deficits in subjects with functional ankle instability. 2005;37(2):169-75.

40. Lin J-Z, Lin Y-A, Tai W-H, Chen C-YJB. Influence of Landing in Neuromuscular Control and Ground Reaction Force with Ankle Instability: A Narrative Review. 2022;9(2):68.

41. Moisan G, Mainville C, Descarreaux M, Cantin VJJoAT. Lower limb biomechanics during drop jump landing in individuals with chronic ankle instability. 2022.

42. Kim H, Son SJ, Seeley MK, Hopkins JTJSjom, sports si. Altered movement strategies during jump landing/cutting in patients with chronic ankle instability. 2019;29(8):1130-40.

43. Riemann BL, Caggiano NAJJoSR. Examination of a Clinical Method of Assessing Postural Control During a Functional. 1999;8:171-83.

44. Ross SE, Guskiewicz KMJIJoAT, Training. Time to stabilization: a method for analyzing dynamic postural stability. 2003;8(3):37-9.

45. Brown CN, Mynark RJJoat. Balance deficits in recreational athletes with chronic ankle instability. 2007;42(3):367.

46. Ross SE, Guskiewicz KM, Gross MT, Yu BJJoat. Assessment tools for identifying functional limitations associated with functional ankle instability. 2008;43(1):44-50.

47. Ross SE, Guskiewicz KM, Yu B. Single-leg jump-landing stabilization times in subjects with functionally unstable ankles. Journal of athletic training. 2005;40(4):298.

48. Simpson JD, Stewart EM, Macias DM, Chander H, Knight ACJPTiS. Individuals with chronic ankle instability exhibit dynamic postural stability deficits and altered unilateral landing biomechanics: A systematic review. 2019;37:210-9.

49. Kim K, Choi B, Lim WJD, Technology RA. The efficacy of virtual reality assisted versus traditional rehabilitation intervention on individuals with functional ankle instability: a pilot randomized controlled trial. 2019;14(3):276-80.

50. Kim KJJJoIAoPTR. Effects of virtual reality programs on proprioception and instability of functional ankle instability. 2015;6(2):891-5.

51. Sharma N, Sharma A, Sandhu JSJAjosm. Functional performance testing in athletes with functional ankle instability. 2011;2(4):249.

52. Woby SR, Roach NK, Urmston M, Watson PJJP. Psychometric properties of the TSK-11: a shortened version of the Tampa Scale for Kinesiophobia. 2005;117(1-2):137-44.

53. Lynall RC, Campbell KR, Mauntel TC, Blackburn JT, Mihalik JPJJoat. Single-legged hop and single-legged squat balance performance in recreational athletes with a history of concussion. 2020;55(5):488-93.

54. Gribble PA, Delahunt E, Bleakley C, Caulfield B, Docherty C, Fourchet F, et al. Selection criteria for patients with chronic ankle instability in controlled research: a position statement of the International Ankle Consortium. 2013;43(8):585-91.

55. Mirshahi M, Halabchi F, Golbakhsh M, Saadat SJAJoEM. Reliability and recalibration of the Persian version of Cumberland Ankle Instability Tool cut-off score in athletes with functional ankle instability. 2019;3(3).

56. Rawcliffe AJ, Hinde KL, Graham SM, Martindale R, Morrison A, Krajewski KT, et al. Altered dynamic postural stability and joint position sense following British Army foot-drill. 2020;2.

57. Rahmati N, Moghadam MAA, Shairi MR, Paknejad M, Rahmati Z, Ghassami M, et al. Psychometric properties of the tampa scale for kinesiophobia amongst iranian patients with chronic persistent pain. 2014;13(2):197-210.

58. Conceição JS, Schaefer de Araújo FG, Santos GM, Keighley J, Dos Santos MJJJoat. Changes in postural control after a ball-kicking balance exercise in individuals with chronic ankle instability. 2016;51(6):480-90.

59. McKeon PO, Ingersoll CD, Kerrigan DC, Saliba E, Bennett BC, Hertel JJM, et al. Balance training improves function and postural control in those with chronic ankle instability. 2008;40(10):1810-9.

60. Hale SA, Hertel J, Olmsted-Kramer LCJJoo, therapy sp. The effect of a 4-week comprehensive rehabilitation program on postural control and lower extremity function in individuals with chronic ankle instability. 2007;37(6):303-11.

61. Bagheri H, Khanmohammadi R, Olyaei G, Talebian S, Hadian MR, Najafi MJNL. Video game and motor-cognitive dual-task training could be suitable treatments to improve dual-task interference in older adults. 2021;760:136099.

1. Ankle Sprain [↑](#footnote-ref-2)
2. Chronic Ankle Instability (CAI) [↑](#footnote-ref-3)
3. Giving Way [↑](#footnote-ref-4)
4. Base of Support (BOS) [↑](#footnote-ref-5)
5. Non-immersive Virtual Reality [↑](#footnote-ref-6)
6. Feedback [↑](#footnote-ref-7)
7. Cognitive Load [↑](#footnote-ref-8)
8. Jump-Landing [↑](#footnote-ref-9)
9. Multiple Single Hop [↑](#footnote-ref-10)
10. Stability Index [↑](#footnote-ref-11)
11. Time to Stabilization [↑](#footnote-ref-12)
12. The Cumberland Ankle Instability Tool (CAIT) [↑](#footnote-ref-13)
13. The Foot and Ankle Ability Measure (FAAM) [↑](#footnote-ref-14)
14. Time Series [↑](#footnote-ref-15)
15. Sequential Averaging [↑](#footnote-ref-16)
16. Single Hop Test [↑](#footnote-ref-17)
17. Side Hop Test [↑](#footnote-ref-18)
