# Supplementary material for: Comparison of the effects of exergaming and balance training on dynamic postural stability during jump-landing in recreational athletes with chronic ankle instability
Source: PLoS One. 2024 Dec 16;19(12):e0314686. doi: 10.1371/journal.pone.0314686 (PMC11649137; doi:10.1371/journal.pone.0314686)
Supplement: Attachment — Submitted filename: response to the original reviewers comments.docx [file pone.0314686.s005.docx]

***Dear Reviewer 1:***

Thank you for carefully reading our manuscript and providing valuable comments and suggestions. We believe the manuscript has significantly improved after incorporating your feedback. We have applied your comments throughout the text, indicated in red.

**Comments**

**Question 1:** **Keywords: Keywords should be reflective of your works. Please edit it. Answer 1:** We modified the keywords to “Virtual Reality Exercise; Chronic Ankle Instability; Exercise Therapy; Balance; Jump”.

**Question 2:** **Methods. Please provide more explanations about translation and psychometrics properties of Persian version of questionnaires used in the study. Answer 2:** We added following sentences to the method section.

**“2.5.3. Tampa Scale for Kinesiophobia (TSK)**

…... The Persian version of TSK-11 has a acceptable internal consistency (Cronbach alpha 0.93) and excellent test-retest reliability (ICC=0.93, 95% CI: 0.92-0.94) [1].

**2.5.4. Cumberland Ankle Instability Tool (CAIT)**

…. The Persian version of this questionnaire has good internal consistency (Cronbach’s α of 0.78 for the right ankle and 0.79 for the left ankle) and substantial reliability (ICC_(2, 1)_ = 0.88; 95% CI: 0.86 – 0.90) in athletes [2].”

1.	Rahmati, N., et al., *Psychometric properties of the Tampa Scale for Kinesiophobia amongst Iranian patients with chronic persistent pain.* J Payesh, 2014. **13**(2): p. 197-210.

2.	Mirshahi, M., et al., *Reliability and Recalibration of the Persian Version of Cumberland Ankle Instability Tool Cut-off Score in Athletes with Functional Ankle Instability.* Adv J Emerg Med, 2019. **3**(3): p. e26.

3.	Wulf, G., C. Shea, and R. Lewthwaite, *Motor skill learning and performance: a review of influential factors.* Med Educ, 2010. **44**(1): p. 75-84.

***Dear Reviewer 2:***

Thank you for carefully reading our manuscript and providing valuable comments and suggestions. We believe the manuscript has significantly improved after incorporating your feedback. We have applied your comments throughout the text, indicated in blue.

**Comments**

**Question 1:** **L. 47. You should change “intervention group” to “exergaming group”.**

**Answer 1:** We changed it.

**Question 2:** **L. 274–275. It would be easier to unify the group names to exergaming and balance training only.**

**Answer 2:** We applied this comment.

**Question 3:** **Statistical analysis: Due to differences in joint tissue flexibility between males and females, it is recommended that sex is added to the covariates and re-analyzed.**

**Answer 3:** We incorporated your comment by adding the following sentence to the Statistical Analysis section. Additionally, the Results section and Table 5 were updated. Overall, although the values were adjusted, the final results and discussion remained the same—meaning that significant values did not become non-significant and vice versa.

“Although the gender distribution was not statistically significant between the groups, it was nearly significant (p=0.067) and could potentially influence the results. Therefore, this variable was considered as a covariate to control for its effect. A two-way mixed ANCOVA with one within-subjects factor and one between-groups factor was conducted to examine the main effects of group (exergaming and balance training), time (before, after, and follow-up) and their interactions.”

**Question 4:** **What is the duration of morbidity (from when is it CAI) of the subjects included in this study? Or what is the total number of sprains?**

**You mention damage to the mechanical receptors due to repeated sprains, but how much damage is caused by repeated sprains?**

**Without this information, it is not possible to determine whether this statement also applies to the present subject.**

**Answer 4:** We added 'time since first sprain (months)' and 'number of ankle sprains' to Table 1 to present the clinical characteristics of the subjects. Additionally, the Cumberland Ankle Instability Tool (CAIT) score, which is presented in Table 1 and explained in section 2.5.4 of the text, reflects the severity of perceived ankle instability**.** This score helps estimate the extent of damage caused by frequent sprains and determines the level of instability.

**Question 5:** **P. 26, L. 110–121. In this paragraph, it is stated that exergaming is more likely to result in a greater number of motor tasks for weight shift in the medial and lateral direction. In that case, the conclusion is not that exergaming is more effective, but that the difference is simply due to differences in learning effects.**

**Answer 5:** To clarify the meaning, we have revised the paragraph as follows:

“The study results indicated that exergaming significantly improved stability in the ML direction compared to balance training, with these effects lasting up to one month post-treatment. This enhancement may be attributed to the emphasis on ML weight shifting in various games, such as single-leg swings, leg raises, table tilts, penguin fishing, soccer heading, tightrope walking, and snowboard slalom. Repeated practice of these ML movements, which demand precise and coordinated control, fosters motor learning. Motor learning involves changes in the nervous system that improve the efficiency and accuracy of specific movements. This process is essential for enhancing postural stability and balance, as it aids individuals in maintaining and controlling posture, adapting to disturbances, and integrating sensory and motor information effectively [3]. Training programs that emphasize motor learning can significantly improve postural stability and overall balance. Consequently, the enhanced stability in the ML direction can be attributed to the role of exergaming in facilitating motor learning and, therefore, improving postural stability.”
