# Supplementary material for: Comparison of the effects of exergaming and balance training on dynamic postural stability during jump-landing in recreational athletes with chronic ankle instability
Source: PLoS One. 2024 Dec 16;19(12):e0314686. doi: 10.1371/journal.pone.0314686 (PMC11649137; doi:10.1371/journal.pone.0314686)
Supplement: Attachment — Submitted filename: Dear Reviewer 3.docx [file pone.0314686.s006.docx]

**Dear Reviewer 3**

We sincerely appreciate your thorough review of the manuscript and the valuable comments and suggestions provided. We believe that the manuscript has significantly improved as a result of incorporating your feedback, which has been highlighted in blue throughout the text.

**Question 1:** Participants inclusion and exclusion criteria should be clearer.

**Answer 1:** We have revised this section as follows.

“**Inclusion Criteria**:

1. Age between 18 and 40 years.
2. A self-reported history of at least one significant acute ankle sprain that:
   - Occurred more than 12 months prior to study enrollment.
   - Was associated with inflammatory symptoms, such as pain and swelling.
   - Resulted in at least one day of interrupted physical activity [2].
3. The most recent ankle sprain occurred more than 3 months before study enrollment [2].
4. A history of at least two episodes of "giving way," recurrent ankle sprains, or "feelings of ankle joint instability" within the last 6 months:
   - "Giving way" refers to uncontrolled and unpredictable episodes of excessive inversion of the rear foot during activities, which do not result in an acute sprain.
   - "Recurrent sprains" are defined as two or more sprains to the same ankle.
   - "Feelings of instability" refer to the perception of instability during daily activities or sports, often associated with the fear of a new ankle sprain [2].
5. A Cumberland Ankle Instability Tool (CAIT) score of ≤ 24 (the CAIT is scored from 0–30, with lower scores indicating more instability) [2].
6. Participants must be recreationally active, engaging in sports that involve jumping (such as volleyball, basketball, soccer, or handball) at least three times a week, for a minimum of 30 minutes per session [27].
7. No self-reported history of fractures or surgery in the lower extremity.
8. No known psychological or neurological disorders.
9. No prior experience with video game-based training.

**Exclusion Criteria**:

1. If participants missed three or more non-consecutive training sessions, or two consecutive sessions.
2. They unable to perform the required maneuvers. »

**Question 2:** Primary outcomes measures and Secondary outcome measures, kindly provide more reason in detail about their selection in this study.
**Answer 2:** We revised this section to “The primary outcome measures included the SI and the TTS in the anteroposterior (AP), mediolateral (ML), and vertical (V) directions, along with the dynamic postural stability index (DPSI) and the resultant vector time to stabilization (RVTTS) during jump landing. The SI and TTS were selected for their strong reliability as established metrics for evaluating dynamic balance during jump landings. These parameters can distinguish individuals with CAI from those who are healthy, as research show that individuals with CAI typically achieve higher scores on the SI and TTS than their healthy counterparts [25]. Furthermore, these parameters are associated with an increased risk of recurrent injuries [26].

These measures enable the evaluation of neuromuscular control, which is crucial in minimizing the risk of re-injury and improving functional outcomes in sports. Thus, improving these parameters and ensuring sufficient stability during jump landings is a vital aspect of the rehabilitation program.

Secondary outcomes included performance metrics, fear of movement, and the severity of perceived ankle instability, assessed using the side-hop test, the Tampa Scale for Kinesiophobia (TSK), and the Cumberland Ankle Instability Tool (CAIT), respectively. These secondary outcomes were selected to provide a more holistic evaluation of the participants' functional and psychological well-being.

The side-hop test measures agility and functional performance of the ankle during dynamic activities, while the TSK evaluates fear of movement or re-injury—an important factor that can significantly impact rehabilitation and athletic performance. The CAIT assesses participants' subjective perceptions of ankle instability, which is essential for understanding their overall ankle function. Incorporating these secondary measures enhances the primary dynamic postural stability outcomes by considering both functional performance and psychological factors that play a crucial role in the rehabilitation process and long-term recovery for athletes with CAI.”

**Question 3:** A force plate device (Bertec Corporation, Columbus, OH, USA)- Reliability and validity of this device is needed.

**Answer 3:** We have incorporated the following sentences to address your comment.

“The Bertec force plate is well-known for its exceptional reliability and validity in assessing ground reaction forces (GRF). Numerous studies have shown that it provides accurate and consistent data across different movement patterns, establishing it as a "gold standard" for evaluating balance and center of pressure (COP) measurements [28-30].”

**Question 4:** The description of games- Can you able to provide the reasons for selecting these specific games.
**Answer 4:** We added the following paragraph to the text

“These games, categorized as balance games in Wii Fit, are primarily intended to improve postural stability. Selected games, including the Single Leg Extension, Torso Twist, Single Leg Twist, Sideways Leg Lift, and Rowing Squat, require participants to maintain balance while executing various movements. This focus on dynamic balance is essential for athletes and individuals recovering from ankle injuries. The games simulate movements and challenges commonly faced in both sports and daily life. Additionally, weight-shifting activities in Snowboard Slalom, Table Tilt, Penguin Fishing, Soccer Heading, and Tightrope Walk closely reflect real-world scenarios, making them particularly relevant for athletes striving to regain dynamic stability and confidence in their performance. Furthermore, many of these games, like Rowing Squat, engage multiple muscle groups, enhancing overall strength, coordination, and endurance. This comprehensive approach is crucial for rehabilitation, effectively preparing participants for the physical demands of their sports and daily activities.”

**Question 5:** English language correction is needed in some parts of this study.

**Answer 5:** The manuscript was reviewed by a native English speaker.
