# Supplementary material for: Comparison of the effects of exergaming and balance training on dynamic postural stability during jump-landing in recreational athletes with chronic ankle instability
Source: PLoS One. 2024 Dec 16;19(12):e0314686. doi: 10.1371/journal.pone.0314686 (PMC11649137; doi:10.1371/journal.pone.0314686)
Supplement: Attachment — Submitted filename: Dear Reviewer 1.docx [file pone.0314686.s007.docx]

**Dear Reviewer 1**

We sincerely appreciate your thorough review of the manuscript and the valuable comments and suggestions provided. We believe that the manuscript has significantly improved as a result of incorporating your feedback, which has been highlighted in red throughout the text.

**Question 1:** 1- Table 1: Check the data for normal distributions. If variables do not follow normal distributions, summarize using median, first and third quartiles and compare using Wilcoxon rank sum test

**Answer 1:** Thank you for your attention to this detail. As previously noted, all variables except for age and time since the last ankle sprain followed a normal distribution. Consequently, we revised Table 1 to present the median and interquartile range (Q1 and Q3) for these two variables in place of the mean and standard deviation.
